# Initial safe(r)-by-design assessment of printable konjac-agar-pectin hydrogels for future extrusion-based bioprinting applications

**DOI:** 10.3389/fbioe.2026.1900478

**Published:** 2026-08-28

**Authors:** Laura Mendoza-Cerezo, Francisco Iñesta‐Vaquera, Antonio Macías-García, Alfonso C. Marcos-Romero, Jesús M. Rodríguez-Rego

**Affiliations:** 1 Departamento de Expresión Gráfica, Escuela de Ingenierías Industriales, Universidad de Extremadura, Badajoz, Spain; 2 Departamento de Bioquímica, Biología Molecular y Genética, Facultad de Ciencias, Universidad de Extremadura, Badajoz, Spain

**Keywords:** 3D bioprinting, biochemistry, cellular response, characterisation, hydrogels, printability

## Abstract

**Introduction:**

Natural polysaccharide‐based hydrogels are promising materials for extrusion‐based 3D bioprinting, although achieving an adequate balance between cellular response, extrudability and shape fidelity remains challenging.

**Methods:**

In this study, hydrogel formulations based on konjac glucomannan, agar and citrus pectin were developed and evaluated under an initial safe(r)‐by‐design approach for future extrusion‐based bioprinting applications. The materials were combined at different concentrations to assess their rheological behaviour, direct‐contact cellular response and printability-related performance.

**Results:**

Konjac glucomannan contributed to water retention and viscosity modulation, agar provided thermal gelation and structural reinforcement, and citrus pectin modified flow behaviour and polymer‐network interactions. Rheological analysis showed shear‐thinning flow for all formulations, while the four selected formulations showed G′ values higher than G″ throughout the 0.1–20 Hz frequency range and re-established G′ > G″ after large‐amplitude deformation. Increasing agar concentration generally increased stiffness and extrusion resistance, whereas pectin reduced resistance to flow and was associated with lower mean relative pore‐area values in the analysed grids. The direct‐contact MTS assay was interpreted as a measure of apparent metabolic activity, rather than as an absolute quantification of viable cell number, due to the possible influence of hydrogel‐assay interactions. Printability‐related assays showed that the pectin‐free formulations, particularly 2K0.1A and 2K0.2A, exhibited higher mean relative pore-area values in the analysed grids, whereas the pectin-containing formulations showed lower extrusion resistance and improved flowability but lower mean relative pore-area values.

**Discussion:**

Overall, 2K0.1A showed the most balanced behaviour among the evaluated formulations when considering extrusion resistance, collapse performance, relative self-supporting index and the descriptive pore-area results. These results support the potential of konjac-agar-pectin hydrogels as printable natural-material platforms, while highlighting the need for further optimisation and validation under cell-laden and tissue-specific conditions.

## 1 Introduction

Bioprinting is an additive manufacturing approach that has gained increasing relevance in biomedical research and regenerative medicine. This technique offers promising possibilities for tissue engineering, controlled drug delivery, and the development of organoid-like models for pharmacological and disease studies. Its fundamental principle is based on the layer-by-layer deposition of biomaterials that mimic certain functions of the extracellular matrix (ECM) and, in future cell-laden applications, can provide a supportive environment for embedded or associated cells. Among these biomaterials, hydrogels are particularly relevant because they form three-dimensional hydrophilic polymer networks capable of absorbing and retaining large amounts of water, while maintaining their structure through chemical or physical crosslinking of polymer chains (Barasinski et al., 2022). These properties make hydrogels useful platforms for recreating hydrated three-dimensional microenvironments with relevance for biomedical applications.

Hydrogels used in bioprinting are commonly classified into natural and synthetic systems. Synthetic hydrogels generally offer high control over composition, mechanical properties and shape fidelity, which facilitates the fabrication of three-dimensional structures with defined geometries (Gyles et al., 2017). However, some synthetic systems may present limitations related to potential cytotoxicity, degradation products or immune response, depending on their composition and crosslinking chemistry (Gul et al., 2022). In contrast, hydrogels derived from natural biopolymers, including polysaccharides and proteins, are often valued for their high water content, biological similarity to extracellular matrices and favourable cytocompatibility profiles (Fan et al., 2022). Nevertheless, their mechanical properties and post-printing stability are frequently lower than those of synthetic hydrogels (Gul et al., 2022). For this reason, hybrid hydrogels are commonly used to combine the advantages of both material families. Even so, the development of natural hydrogel systems with improved balance between biological response, extrudability and structural stability remains an important research objective.

Natural hydrogels have gained considerable relevance as base materials for bioprinting because of their high-water content, biodegradability and biological similarity to extracellular matrix components. Several natural or naturally derived hydrogel systems have been explored for this purpose. For example, alginate-gelatin systems, such as that developed by Kim et al., have shown potential to support osteogenic behaviour in encapsulated cells (Kim et al., 2023). Gelatin methacrylate (GelMA) hydrogels have also been extensively used because of their controllable photocrosslinking and their ability to provide a favourable environment for cell culture, as reported by Tanadchangsaeng et al. using fish-derived gelatin for skin replacement applications (Tanadchangsaeng et al., 2024). Likewise, kappa-carrageenan-gelatin formulations have demonstrated structural and mechanical stability for wound-healing applications (Wang et al., 2025), while chitosan-gellan gum hydrogels, such as those developed by Robinson et al., have been used to generate robust and aligned microstructures (Robinson et al., 2020). In addition, nanocellulose-alginate hydrogels have shown good shape fidelity and suitable cell viability levels (Jessop et al., 2019).

Despite these advances, many hydrogel systems used in extrusion-based bioprinting require chemical, ionic or photoinduced crosslinking steps to achieve sufficient mechanical stability after deposition. Depending on the material system and the crosslinking strategy used, these additional steps may introduce variables that affect cell response, hydrogel structure, degradation behaviour or reproducibility. Therefore, there is continued interest in developing hydrogel formulations capable of achieving adequate rheological, biological and printability-related properties while reducing the need for external crosslinking agents during the printing process.

In the present study, hydrogels were formulated using agar-agar, konjac glucomannan and citrus pectin, three naturally derived polysaccharides widely used in the food and biomedical-related fields and with physicochemical properties potentially transferable to extrusion-based bioprinting. These components were selected because they can contribute complementary properties to the final hydrogel system: konjac glucomannan provides high water-retention capacity, agar contributes to thermal gelation and structural reinforcement, and citrus pectin may modulate flow behaviour and polymer-network interactions. The resulting formulations were designed to form physically stabilised hydrogel networks without requiring additional chemical crosslinking during printing, which may simplify material preparation and reduce the number of processing variables that could affect cellular response or printing performance.

Konjac glucomannan, obtained from the tuber of *Amorphophallus konjac*, is a water-soluble polysaccharide with abundant hydroxyl and carbonyl groups, composed of D-mannose and D-glucose units linked by β-(1→4) bonds, with acetyl substituents along the polymer chain (Yang et al., 2017). Its gelation behaviour has been associated with deacetylation-dependent chain aggregation, in which polymer chains can adopt random-coil conformations and subsequently associate through intermolecular interactions to form a gel network (Sun et al., 2023). This polymer is also known for its high water-retention capacity and its ability to form viscous aqueous systems at relatively low concentrations (Tong et al., 2022). In the present work, konjac glucomannan was incorporated as the main water-retaining and viscosity-modulating component of the hydrogel formulations, in combination with agar and pectin.

Agar is a complex polysaccharide composed of repeating units linked by β-(1,4) and α-(1,3) glycosidic bonds (Jeong et al., 2018). It is obtained from red algae, including species of the genera *Gelidium*, *Eucheuma* and *Gracilaria*, and is mainly composed of agarose, which is primarily responsible for gel formation, and agaropectin, which contributes to its physicochemical behaviour (Rahman et al., 2020). Agar is widely recognised for its thermoreversible gelation, with melting temperatures generally above 95 °C and setting temperatures between 10 °C and 40 °C (Lahaye et al., 1991). During cooling, agarose chains form ordered helical structures stabilised by hydrogen bonding, generating a three-dimensional network capable of retaining large amounts of water (Pandya et al., 2022). In the present formulations, agar was incorporated as the main component contributing to thermal gelation and structural reinforcement.

Pectin is one of the most complex polysaccharides present in plant cell walls and is mainly composed of galacturonic acid units arranged in different structural domains, including homogalacturonan, rhamnogalacturonan I and rhamnogalacturonan II (Said et al., 2023). Its gelation behaviour depends strongly on its degree of methoxylation and on the surrounding physicochemical conditions. High-methoxyl pectins can form gels under acidic conditions and in the presence of high soluble-solid content, whereas low-methoxyl pectins can form ionically crosslinked networks through calcium-mediated interactions (Rivera-Hernández et al., 2023). Beyond these classical gelation mechanisms, pectin is also relevant in biomedical applications because of its water-retention capacity, acidic character and affinity for bioactive molecules, including growth factors (Syn et al., 2020). Pectin-based hydrogels have therefore been explored as biomaterials with favourable cytocompatibility and tunable mechanical behaviour (Belousov et al., 2022). In the present formulations, citrus pectin was incorporated not as an independently crosslinked gel-forming component, but as a polymeric additive intended to modulate flow behaviour, network interactions and the balance between extrudability and structural definition.

Agar, konjac glucomannan and pectin have been previously reported as materials with low cytotoxicity or favourable cytocompatibility profiles in different experimental contexts (Kanniyappan et al., 2020), (Hu et al., 2023). However, their biological response cannot be assumed to be identical when they are combined into a new ternary hydrogel system, since polymer concentration, network structure, sterilisation conditions and the interaction with cells may influence the final cellular response. Therefore, experimental evaluation of the complete formulations remains necessary.

In addition to cellular response, rheological behaviour is a critical factor for extrusion-based bioprinting, since viscosity, shear-thinning response and post-deposition stability strongly influence filament formation and shape retention during printing (Qiao et al., 2022). Konjac-based hydrogels may present limitations when used alone, including high viscosity, excessive hydrophilicity and insufficient mechanical stability for maintaining printed structures (Qiao et al., 2022). The combination of konjac with agar has been proposed as a strategy to improve gel stability and mechanical performance (Qiao et al., 2023), while the incorporation of pectin may further modulate the balance between flowability, network cohesion and biological response.

Previous studies have explored binary combinations of these polysaccharides, such as agar-konjac, agar-pectin and konjac-pectin systems, although generally for applications outside the specific context of extrusion-based bioprinting. Agar-konjac systems have been investigated because of their uniform porous structure, large surface area and improved peptide adsorption capacity (Min et al., 2025). Agar-pectin mixtures have also been studied in the field of cultural heritage restoration, where they have shown the ability to form homogeneous, transparent and self-supporting gels with more controlled water and ethanol absorption compared with pure agar (Spitz-Argolo et al., 2025). In addition, konjac-pectin systems have been applied in wastewater treatment and phosphorus recovery from livestock effluents (Wu et al., 2023).

However, to the best of our knowledge, the combined use of agar, konjac glucomannan and citrus pectin as a ternary hydrogel system has not been systematically evaluated in the specific context of extrusion-based bioprinting. In particular, the combined effect of these three components on rheological behaviour, extrusion performance, shape fidelity and cellular response remains insufficiently characterised. This study addresses this gap by providing an integrated initial assessment of konjac-agar-pectin formulations under a safe(r)-by-design approach, in which material processability and cellular response were evaluated before progressing towards future cell-laden printing studies.

Hydrogels based on konjac glucomannan, agar and citrus pectin may represent a promising natural-material platform because their physical network formation can reduce the need for additional chemical crosslinking steps during printing. Since no standardised natural hydrogel formulation currently fulfils all the biological, rheological and printability requirements for every bioprinting application, further exploration of new polymer combinations remains necessary. In this context, the present work evaluates formulations with different proportions of konjac, agar and citrus pectin in terms of rheology, direct-contact cellular response and printability-related behaviour. The aim was to identify formulations with a favourable balance between extrusion behaviour, short-term structural performance, shape fidelity and preliminary cellular response, as a basis for future optimisation and validation in cell-laden extrusion-based bioprinting applications.

## 2 Materials and methods

### 2.1 Preparation of the different hydrogel formulations

The hydrogel components were initially prepared as independent solutions, each dissolved in 100 mM Tris-HCl buffer (pH ≈ 7), for subsequent sterilisation and cellular-response screening at different concentrations (Table 1). Konjac glucomannan was dispersed in Tris-HCl buffer and stirred mechanically at 1000 rpm for 15 min at 85 °C. Agar was dissolved separately under magnetic stirring at 500 rpm for 30 min at 95 °C, while citrus pectin was maintained at 85 °C under magnetic stirring at 500 rpm for 15 min.

**TABLE 1:** Concentrations (w/v) of the hydrogel components used in initial direct-contact MTS screening.

Konjac	Agar-agar	Citrus pectin
3	0.5	2.5
2.5	0.4	2
2	0.3	1.5
1.5	0.2	1
​	0.1	0.5

For the preparation of the composite hydrogel, agar was first diluted to the desired concentration and kept under stirring at 500 rpm for 30 min at 95 °C until complete dissolution. Pectin powder was then added, maintaining the same temperature and stirring conditions until a homogeneous mixture was obtained. Finally, the agar-pectin mixture was subjected to mechanical stirring at 1000 rpm, with konjac powder slowly added until complete dissolution after 30 min of mixing. The resulting solutions were sterilized by autoclaving (121 °C, 20 min) and stored at 4 °C until use. Prior to rheological and bioprinting tests, the formulations were equilibrated at 37 °C for 30 min to ensure thermal homogeneity and reproducibility.

The formulations developed in this work did not require chemical crosslinking during preparation or printing, as the polysaccharide combinations formed physically stabilised networks with sufficient short-term cohesion for rheological characterisation and printability-related assays under the selected experimental conditions.

### 2.2 Rheology

The rheological properties of the hydrogel formulations were characterised using a Kinexus Prime Pro + rotational rheometer (NETZSCH, Germany) equipped with a temperature-control system. Measurements were performed using a 40 mm parallel-plate geometry with a fixed gap of 0.4 mm. After loading each sample, the excess material was carefully trimmed to avoid edge artefacts. The samples were then allowed to equilibrate for 15 min at the corresponding test temperature before measurement. All rheological experiments were performed in triplicate for each formulation.

A series of rheological tests was carried out to evaluate the linear viscoelastic range, temperature-dependent viscoelastic response, frequency-dependent behaviour, flow properties and recovery after structural disruption.

First, an amplitude sweep test was performed to determine the linear viscoelastic range (LVR). The test was conducted at 37 °C and 1 Hz, increasing the complex shear strain from 0.01% to 100%. The storage modulus (G′) and loss modulus (G″) were recorded as a function of strain. The LVR was defined as the strain region in which G′ remained approximately constant and independent of the applied deformation. Based on this test, a strain value of 0.5% was selected for subsequent oscillatory measurements, as it was within the LVR of the representative formulations analysed. Although the LVR extended up to approximately 10% strain in the representative formulations, 0.5% strain was selected as a conservative value to minimise structural perturbation during subsequent small-amplitude oscillatory measurements.

Second, temperature sweep tests were performed to assess the thermal stability of the hydrogels under conditions close to those used during printing and biological evaluation. The temperature was increased from 25 °C to 40 °C while recording G′ and G″ at a fixed frequency of 1 Hz and a strain of 0.5%. This test was used to identify possible thermally induced changes in the internal structure of the hydrogels and to verify whether the formulations maintained a predominantly solid-like behaviour near physiological temperature.

Third, frequency sweep tests were performed on the four formulations selected for the final printability assessment: 2 K0.1A, 2K0.1A1P, 2 K0.2A and 2K0.2A1P. Measurements were conducted at 37 °C over a frequency range from 0.1 to 20 Hz, using a strain amplitude of 0.5%, which was within the linear viscoelastic region. Frequencies were distributed logarithmically using 10 points per decade. The storage modulus (G′) and loss modulus (G″) were recorded as functions of frequency. Three independent measurements were performed for each formulation using a separately loaded sample.

Fourth, flow tests were performed to determine the apparent viscosity of the formulations as a function of shear rate. The shear rate was increased from 0.1 to 10,000 s^-1^ at 37 °C. This range was selected to evaluate the shear-thinning behaviour of the hydrogels under conditions relevant to extrusion-based 3D printing. Apparent viscosity curves were represented in log-log scale, as this format allows clearer comparison of the non-Newtonian behaviour of the different formulations.

Unless otherwise specified, small-amplitude oscillatory measurements were performed at 0.5% strain, which was previously confirmed to be within the LVR. The rheological analysis was used to compare the effects of konjac, agar and pectin concentration on hydrogel stiffness, temperature- and frequency-dependent viscoelastic behaviour, extrusion-related flow properties and recovery after structural disruption.

A three-step oscillatory strain-recovery test was performed on the four formulations selected for the final printability assessment: 2 K0.1A, 2K0.1A1P, 2 K0.2A and 2K0.2A1P. Measurements were conducted at 37 °C and a fixed frequency of 1 Hz under strain-controlled oscillation. During the first interval, the samples were subjected to 0.5% strain for 60 s to establish the initial viscoelastic response within the linear viscoelastic region. The strain was then increased to 300%, selected because it was outside the linear viscoelastic region and produced a marked decrease in G′ together with an inversion to G″ > G′, for 60 s to disrupt the viscoelastic structure. Finally, the strain was returned to 0.5% for 300 s to monitor the recovery of the storage modulus (G′) and loss modulus (G″). Three independent measurements were performed for each formulation using a separately loaded sample.

A complementary three-step shear-rate recovery test was also performed at 37 °C on the same four selected formulations. During the first interval, a shear rate of 0.1 s^-1^ was applied for 60 s to record the initial apparent viscosity. The shear rate was subsequently increased to 100 s^-1^ for 60 s and then returned to 0.1 s^-1^ for 280 s. Apparent viscosity was recorded throughout the three intervals to assess the recovery of flow-related viscosity when the initial low-shear condition was restored. Three independent measurements were performed for each formulation using a separately loaded sample.

### 2.3 Printability

Printability was evaluated as a combination of complementary extrusion-related and shape-fidelity assays, rather than as a single parameter. In this study, printability was defined as the ability of a hydrogel formulation to be extruded through a standard bioprinting nozzle under controlled conditions, to form continuous filaments, and to preserve its geometry after deposition. Accordingly, five complementary analyses were performed: minimum extrusion force, collapse behaviour, relative self-supporting index, filament line uniformity, and grid-shape fidelity.

All printability experiments were performed using sterile conical nozzles (Nordson EFD, tapered tips, 22G, inner diameter ≈0.41 mm), mounted on the BIO X bioprinter at 37 °C, unless otherwise specified. This nozzle gauge was chosen as a standard size commonly used for extrusion-based bioprinting and maintained constant across all formulations to ensure comparability.

#### 2.3.1 Extrusion force assay

**FIGURE 1:**
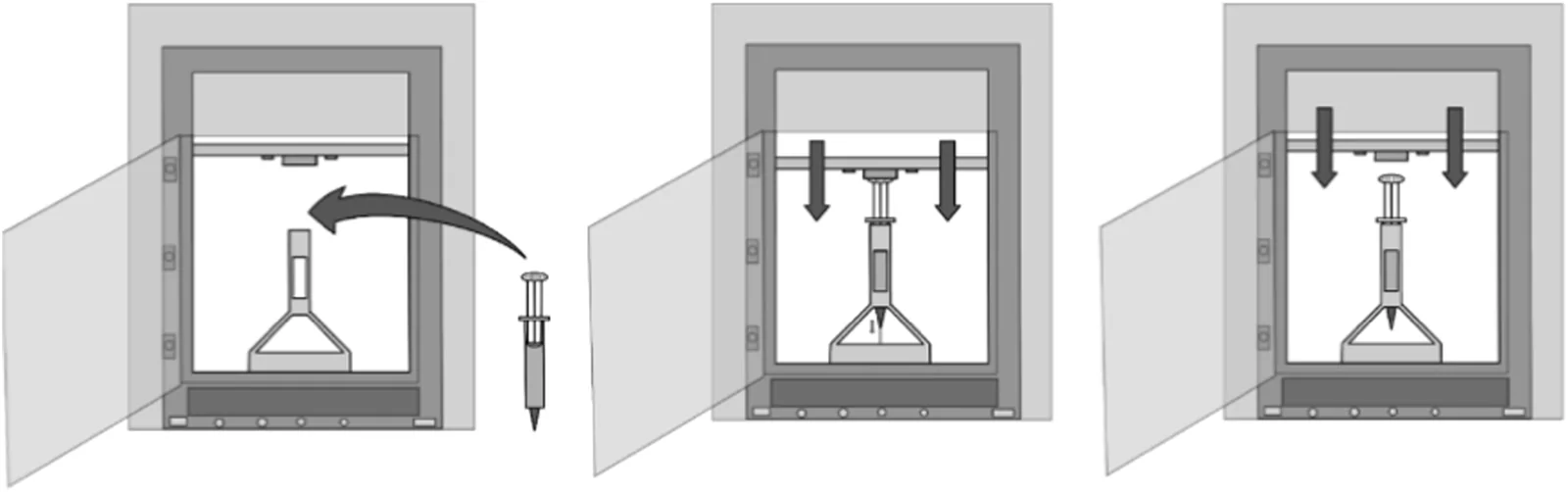
Process of loading the bioink cartridge to perform the extrusion force assay.

The extrusion force assay was performed using an Instron 6800 universal testing system equipped with a 100 N load cell and a custom-designed device to maintain the hydrogel at 37 °C. The cartridge plunger was displaced at a crosshead speed of 5 mm/min. The minimum force required for initiating and maintaining stable extrusion was obtained by recording the resistance exerted by the piston of a cartridge full of hydrogel during compression at constant speed, until the force values reached a stable plateau (Figure 1).

This assay was used as a comparative method to determine the minimum force required for each formulation under identical cartridge, nozzle, temperature and compression conditions.

#### 2.3.2 Collapse test

The platform for carrying out the collapse test was designed using Autodesk® Inventor® software and printed in PLA with the Ender 3 printer.

The collapse test was used to evaluate the ability of the extruded hydrogel filaments to span unsupported gaps between pillars. This assay was considered a shape-retention test related to printability, since it provides information on the capacity of the deposited filament to resist deformation immediately after extrusion.

Filaments of each formulation were extruded with the bioprinter to test their ability to form bridges. The number of supported pillars was used as comparative indicators of filament stability.

#### 2.3.3 Relative self-supporting index

The relative self-supporting index was evaluated for the selected hydrogel formulations to provide a comparative estimate of their resistance to the weight of subsequently deposited layers during the construction of multilayer structures in the Z direction. This assay was designed as a comparative test to assess the resistance of each formulation to vertical deformation under compression, rather than as a standardised mechanical compression test.

Compression measurements were performed using an Instron 6800 universal testing system equipped with a 2530-5 N static load cell with a capacity of ±5 N. The load-measurement accuracy was ±0.5% of the reading over the range from 0.02 to 5 N and ±1.0% of the reading over the range from 0.01 to 0.02 N. The mean force values reported in this study ranged from 0.02 to 0.13 N.

For each measurement, approximately 0.5 mL of hydrogel was deposited onto the lower compression plate of a universal testing machine, forming a hydrogel deposit with an initial height of approximately 8 mm and a diameter of approximately 9 mm. The initial height of each sample was measured before compression. The hydrogel was then compressed uniaxially at a crosshead speed of 1.5 mm/min until reaching 20% and 40% deformation relative to its initial height. The compressive force required to reach each deformation level was recorded.

The force values obtained at 20% and 40% deformation were used as relative indicators of the ability of the material to resist vertical deformation under loading. To provide an approximate and comparative estimation of layer-bearing capacity, the force recorded at each deformation level was divided by the estimated weight of a single deposited hydrogel layer. In this study, the estimated weight of one deposited hydrogel layer was 0.0025 N, calculated from the approximate deposited volume and assuming a hydrogel density close to that of water (Equation 1):

$$\text{Relative self}-\text{supporting index}=\frac{\text{compressive}\;\text{force}\;\text{at}\;\text{defined}\;\text{deformation}}{\text{estimated}\;\text{weight}\;\text{of}\;\text{one}\;\text{deposited}\;\text{layer}}$$ (1)

The resulting value should not be interpreted as an absolute prediction of the maximum printable build height, since tolerating 20% or 40% deformation does not necessarily imply preservation of high print fidelity. Instead, this parameter was used as a comparative index to rank the selected formulations according to their resistance to vertical compression under identical testing conditions.

All tests were performed at 37 °C, using the same deposition procedure and compression conditions for all formulations. Measurements were performed in triplicate for each formulation, and results are expressed as mean ± standard deviation. The reported mean force range of 0.02–0.13 N was within the specified load-measurement range. However, because the lowest mean value coincided with the lower boundary of the ±0.5% accuracy range, small numerical differences were interpreted cautiously and together with the experimental dispersion.

This assay provides comparative information on the resistance of the hydrogel formulations to vertical deformation during multilayer extrusion-based 3D printing. Hydrogels showing higher compressive force values at defined deformation levels are expected to better resist vertical deformation during layer-by-layer deposition, whereas formulations with lower values may undergo earlier deformation during the construction of taller printed structures. However, this assay does not replace direct multilayer printing tests and was interpreted only as a relative mechanical screening tool.

#### 2.3.4 Line study

This analysis was performed to assess the homogeneity and dimensional regularity of the extruded hydrogel filaments. Deviations from an ideal uniform filament were quantified after extrusion through a nozzle of known internal diameter.

For each formulation, straight filaments of 35 mm length were extruded using a BIO X bioprinter equipped with a sterile 22G conical nozzle, with an inner diameter of approximately 0.41 mm. Printing was performed at 37 °C, using a printing speed of 9 mm/s. The nozzle-to-substrate distance was fixed at 0.2 mm, corresponding to the selected layer height. Extrusion was pressure-controlled rather than flow-rate-controlled; therefore, no fixed volumetric flow rate was imposed. Instead, the pneumatic pressure was individually adjusted for each formulation to obtain continuous filament deposition under the selected printing conditions.

After extrusion, the deposited filaments were analysed using AutoCAD. The maximum and minimum filament heights were measured along each printed line to evaluate filament regularity. From these measurements, the corresponding rectangular areas formed between each measured point and the substrate were calculated, allowing the comparison of filament uniformity among formulations (Strauß et al., 2021).

For each formulation, a minimum of three filaments per sample were analysed. Minimum and maximum values corresponded to the extreme values detected along the filament profile, whereas mean values and standard deviations were calculated from the measurements obtained along the analysed filaments. The comparison between minimum and maximum values provided information on the dimensional range observed along the filament profile, whereas the mean ± SD values described the overall dispersion of the analysed measurements.

#### 2.3.5 Printability study

The final printability study was designed to evaluate the ability of the selected formulations to reproduce predefined grid-like geometries after extrusion. This analysis was considered a shape-fidelity assessment and was interpreted together with the extrusion-force, collapse, self-supporting and line-uniformity assays.

To do this, filaments of each formulation were extruded with the BIO X bioprinter, adjusting the parameters to the values provided by the previous tests and analysed using computer-aided design software (AutoCAD). The methodology employed allowed several key variables to be analysed:

- Filament profile analysis: the maximum and minimum height of the filament were measured, calculating the area of the rectangles generated from each point to the base. These differences were then compared to evaluate the regularity of the filament.
- Collapse capacity: the structural stability of the hydrogel filaments was evaluated by extruding them at constant speed through a specially designed platform with pillars arranged at different distances, analysing the capacity of the hydrogel to form self-sustaining bridges between the pillars without collapsing.
- Printability: top-view images of the printed grids were acquired and scaled in AutoCAD using a known reference distance included in each image, allowing direct area measurements on the calibrated image. Twenty pore geometries with different theoretical areas were evaluated from one independent printed grid per formulation. The internal contour of each pore was manually traced, and no automated thresholding or segmentation procedure was applied. A pore was considered measurable when a clearly defined internal contour could be identified and reliably traced. When no reliable internal contour could be identified because the pore geometry was not successfully formed or could not be reliably delineated, the result was classified as not assessable (NA).

For each measurable pore, the relative pore area was calculated by dividing the measured internal pore area by the corresponding theoretical CAD area and multiplying by 100 (Equation 2):

$$Relative\;pore\;area\;\left(\%\right)=\frac{measured\;pore\;area}{theoretical\;pore\;area}\times 100$$ (2)

NA values were excluded from the calculation of the mean and standard deviation but were included when reporting the number and percentage of measurable pores. Values above 100% were retained and indicate that the measured internal pore area was larger than the corresponding theoretical area. Results are expressed as mean ± SD of the measurable pore geometries, together with the number of measurable and non-assessable pores. Because the analysed pores had different theoretical sizes and were obtained from one independent printed grid per formulation, the SD represents within-grid dispersion among different pore geometries rather than repeatability among identical pores or independent printing experiments. The trabecular bone-inspired structures were considered qualitative examples of multilayer printability and were not included in the quantitative area analysis.

Because this metric is based on two-dimensional image analysis, small differences between formulations should be interpreted cautiously and together with the variability of the measurements. Demonstrative complex structures, when included, were considered qualitative examples of printability and not quantitative validation of shape fidelity.

### 2.4 Cell culture

MCF-7, RAW 264.7 and Caco-2 cell lines were acquired from the Biomedical Research Support Service at the University of Extremadura and maintained in DMEM high glucose GlutaMAX (Gibco) culture medium supplemented with 10% fetal bovine serum (Gibco) and 1% penicillin-streptomycin (ThermoFisher). Cells were kept at 37 °C, 95% humidity and 5% CO_2_ with periodic medium changes until reaching subconfluence. Subcultures of MCF-7 and Caco-2 were carried out by trypsinisation with trypsin-EDTA (Gibco) and maintained in 75 cm^2^ flasks. Subcultures of RAW 264.7 cells were carried out by scraping for subsequent resuspension in 75 cm^2^ flasks. Triplicate 10,000 cells/well were seeded in 96-well plates and direct-contact MTS assays were performed after 24 h or 48 h incubation.

### 2.5 Direct-contact MTS assay

30 µL of each hydrogel formulation were dispensed into the bottom of 96-well plates to form thin layers. Subsequently, 10,000 cells in 100 µL of complete medium were seeded on top of each layer and cultured for 24 or 48 h. This experimental configuration was designed as a direct-contact screening assay to evaluate the cellular response to the hydrogel surface under culture conditions, rather than as an extract-based cytotoxicity test. Apparent cellular metabolic activity was assessed using the CellTiter 96® AQueous One Solution Cell Proliferation Assay (MTS, Promega), following the manufacturer’s instructions. Briefly, 20 µL of reagent were added to 100 µL of culture medium in 96-well plates and incubated for 4 h before measuring absorbance at 490 nm. Absorbance values were first corrected by subtracting the background signal from acellular gel wells and subsequently normalised to the viability of cells cultured on tissue-culture plastic (set as 100%).

Because the cells were cultured directly on hydrogel layers and the absorbance was measured in the presence of the material, the MTS readout was interpreted as an indicator of apparent metabolic activity under each experimental condition, not as an absolute measurement of viable cell number. Possible matrix-related effects, including reagent diffusion through the hydrogel, formazan recovery, optical path length differences, light scattering, and changes in cell adhesion or aggregation, were therefore considered when interpreting the results.

### 2.6 Microscopy analysis

Confocal imaging was used to qualitatively assess cell morphology, distribution and membrane integrity. 100 μL of the previously described hydrogel formulations were deposited into the bottom of each well of Ibidi µ-Slide 8 Well chambered slides (Ibidi GmbH, Martinsried, Germany). 50,000 cells per well were then seeded either without hydrogel (control) or on top of these hydrogel layers and incubated for 24 h. Subsequently, cultures were stained for 30 min with Hoechst 33342, used to identify cell nuclei, and propidium iodide, used to detect cells with compromised membrane integrity. Hoechst 33342 and propidium iodide were used at final concentrations of 5 μg/mL and 10 μg/mL, respectively. Samples were imaged using an Olympus Fluoview FV1000 confocal microscope equipped with a ×10 objective.

Confocal microscopy was used as a complementary qualitative analysis to the MTS assay, providing information on cell distribution, aggregation, morphology, and membrane integrity. Since no automated segmentation or quantitative image analysis was performed in this study, the microscopy data were not used to calculate absolute viability percentages. Instead, they were interpreted qualitatively to identify whether the decrease in MTS signal was accompanied by evident membrane damage or by changes in cell organisation on the hydrogel surface.

### 2.7 Statistical analysis

Statistical analysis of the MTS data was performed using one-way analysis of variance (ANOVA) followed by Dunnett’s multiple-comparison test against the corresponding untreated control, using Jamovi v.2.7. Analyses were performed separately for each hydrogel component and exposure time and, for the selected formulations, separately for each cell line. Data are presented as mean ± standard deviation (SD).

## 3 Results and discussion

Several recent studies have adopted a methodological approach similar to that of the present work, combining rheological, printability, and biocompatibility assessments to evaluate new natural-based hydrogels for bioprinting. For instance, Ng et al. investigated the influence of starch on the rheology of a gellan gum-collagen hydrogel and evaluated its printability (2D scaffold fabrication), viscoelastic behaviour (amplitude and frequency sweeps), and cell viability (MTS assay and confocal microscopy with F-actin staining) (Ng et al., 2023). Likewise, Butler et al. analyzed the rheology (amplitude sweeps), 2D printability, and biocompatibility (DAPI/PI) of new N,O-carboxymethyl chitosan-agarose hydrogel for 3D bioprinting (Butler et al., 2021). Similarly, Geevarghese et al. studied an alginate-carboxymethyl cellulose-GelMA hydrogel, characterising its print fidelity, spatial resolution, structural stability, rheology (strain and frequency sweeps, thixotropy), and biocompatibility (AlamarBlue and live/dead assays) (Geevarghese et al., 2025). Finally, Ribezzi et al. characterized a methylcellulose-alginate-gelatin A hydrogel using rheological (amplitude sweeps), printability (shape fidelity, uniformity), mechanical (compression, weight stability), and biological (Live/Dead, AlamarBlue) analyses (Ribezzi et al., 2023).

In line with this framework, the present study integrates rheological and printability analyses with direct-contact MTS screening and qualitative confocal assessment of cell morphology and membrane integrity to identify candidate formulations for extrusion-based bioprinting.

### 3.1 Direct-contact assessment of apparent metabolic activity

To further optimize bioink composition, we first evaluated the cellular response to individual hydrogel components using a direct-contact MTS screening assay. Initially, we selected concentrations of konjac and pectin previously published as biocompatible (Alves et al., 2020), (Li et al., 2021). Consequently, low concentrations were selected to avoid compromising the cell adhesion capacity of the hydrogel, while leveraging agar-agar’s ability to impart mechanical and structural stability to the hydrogel. This ensures its integrity during the bioprinting process and its maintenance in the cellular environment. To ensure that observed results reflect mainly on the effects of hydrogel composition on cells rather than other stressors associated with the bioprinting process (e.g., pressure, temperature), we employed a model in which cells are seeded on top of the hydrogel matrix.

**FIGURE 2:**
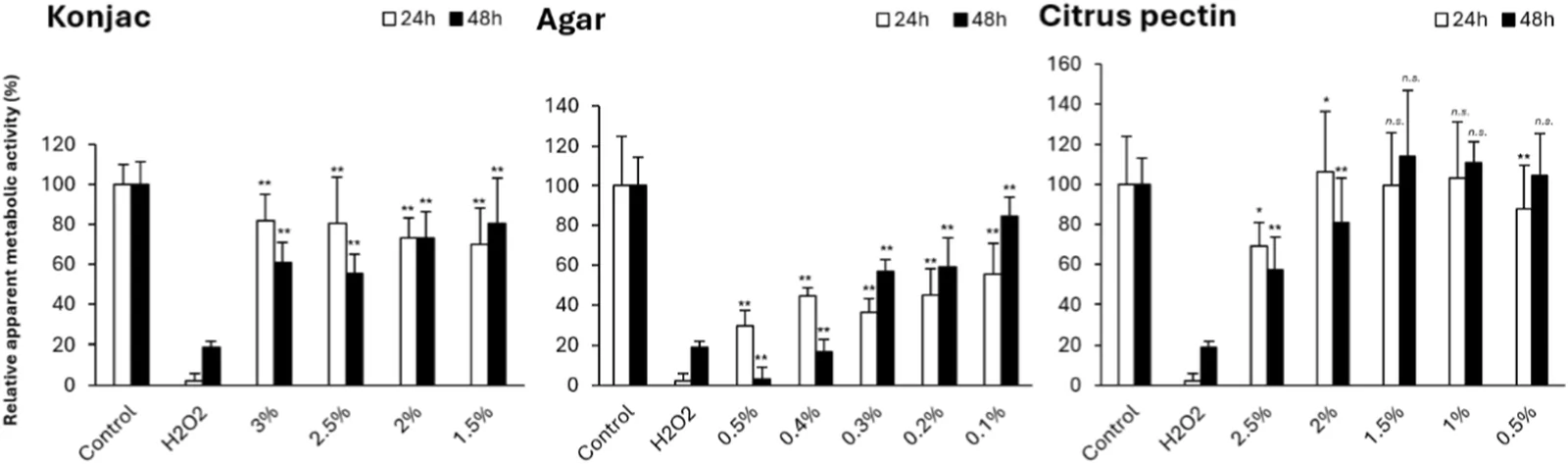
Effect of individual hydrogel components on the apparent metabolic activity of MCF-7 cells. Relative MTS signal after 24 and 48 h of culture in the presence of the indicated components and concentrations. Values were normalised to cells cultured on tissue-culture plastic, set as 100%. Data are presented as mean ± standard deviation of six measurements (n = 6). Statistical significance was analysed separately for each component and exposure time using one-way ANOVA followed by Dunnett’s multiple-comparison test against the corresponding untreated control. *p < 0.05; **p < 0.01; n. s., non-significant. H_2_O_2_ was included as a positive control and was not included in the statistical comparisons.

We first measured the apparent metabolic activity of MCF-7 cells in the absence or presence of decreasing concentrations of bioink components (Figure 2). The hydrogels were formulated at varying concentrations (0.5%–3%), included in 96-well plates and cells were incubated for 24 or 48 h before MTS assessment. We observed a distinct effect of individual hydrogel components on the MTS signal. Konjac glucomannan and agar-agar reduced the apparent metabolic activity of MCF-7 cells, with the effect generally being more pronounced after 48 h. However, these results should not be interpreted exclusively as direct cytotoxicity, since the direct-contact configuration may also reflect reduced cell adhesion, changes in cell morphology or aggregation, and possible matrix-related interference with the MTS readout. The MTS signal decreased markedly in the presence of konjac and agar, with statistically significant differences observed across the tested concentrations. Citrus pectin produced a more moderate and concentration-dependent response. At 48 h, concentrations up to 1.5% showed no statistically significant differences from the control, whereas 2% and 2.5% significantly reduced the MTS signal. At 24 h, significant differences were observed at 0.5%, 2% and 2.5%.

Figure 2 shows the relative MTS metabolic signal after 24 and 48 h of exposure to different concentrations of the individual hydrogel components.

In summary, konjac and agar markedly reduced the MTS metabolic signal at both exposure times, whereas citrus pectin produced a more moderate and concentration-dependent response. Taken together, these results indicate that the cellular response to these components is strongly dependent on polymer type, concentration, and exposure time. Nevertheless, because the MTS assay was performed in direct contact with hydrogel layers, the observed decrease in signal should be interpreted as a preliminary indicator of reduced apparent metabolic activity rather than as definitive evidence of cell death. This distinction was further explored using confocal microscopy to assess cell morphology, distribution, and membrane integrity.

### 3.2 Rheology

Based on the initial biological screening, twelve hydrogel formulations of varying composition, with and without pectin, were developed and are listed in Table 2:

**TABLE 2:** Composition of the different hydrogel formulations evaluated in the rheological and printability studies.

​	Konjac	Agar-agar		ID
Pectin-free hydrogel	1.5	0.1		1.5K0.1A
	1.5	0.2		1.5K0.2A
	2	0.1		2K0.1A
	2	0.2		2K0.2A
​	Konjac	Agar-agar	Citrus pectin	ID
Hydrogels with pectin	2	0.1	1	2K0.1A1P
	2	0.1	1.5	2K0.1A1.5 P
	1.5	0.1	1	1.5K0.1A1P
	1.5	0.1	1.5	1.5K0.1A1.5 P
	2	0.2	1	2K0.2A1P
	2	0.2	1.5	2K0.2A1.5 P
	1.5	0.2	1	1.5K0.2A1P
	1.5	0.2	1.5	1.5K0.2A1.5 P

The rheological behaviour of hydrogels is a crucial factor in assessing their suitability for extrusion-based bioprinting, since it influences material flow through the nozzle, filament formation and the ability of the deposited material to retain its geometry after extrusion. To establish the working LVR and ensure the reliability of subsequent oscillatory measurements, an amplitude sweep test was performed at 37 °C using two representative formulations, 2 K0.1A and 1.5K0.1A1.5 P. These formulations were selected as examples of different rheological responses within the range of compositions evaluated.

**FIGURE 3:**
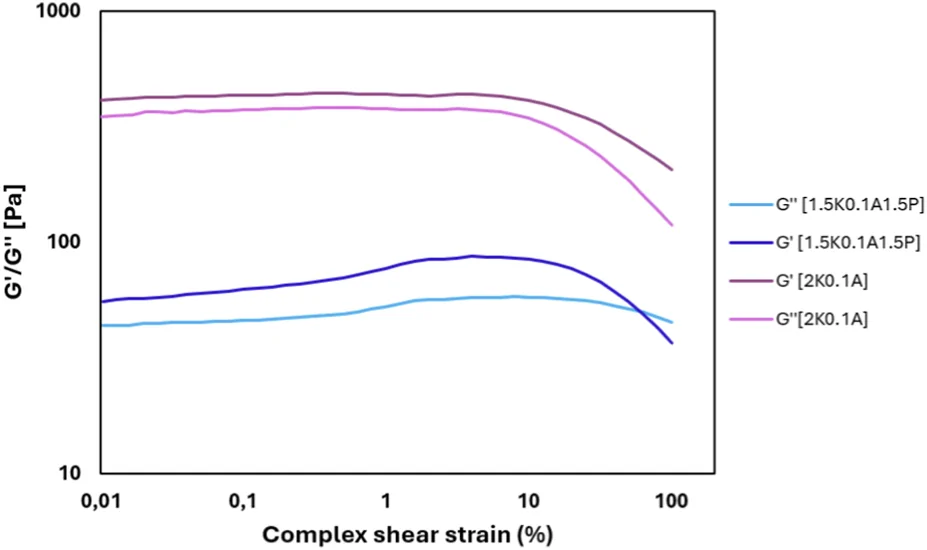
Amplitude sweep characterisation of two representative hydrogels at 37 °C and 1 Hz, used to define the LVR. Storage modulus (G′) and loss modulus (G″) are shown for 2 K0.1A and 1.5K0.1A1.5 P and are expressed in Pa.

The amplitude sweep (Figure 3) showed that the representative formulations maintained an approximately linear viscoelastic response up to around 10% strain, which was adopted as the upper limit of the LVR under the selected conditions. The LVR was defined as the region in which G′ remained approximately constant, with variations below 5% as strain increased. A strain of 0.5% was subsequently selected as a conservative value within the LVR to minimise disruption of the hydrogel network during subsequent small-amplitude oscillatory measurements.

Rheological characterisation of the complete formulation set included temperature-dependent oscillatory measurements and flow tests, whereas frequency-dependent and recovery behaviour were subsequently evaluated for the four formulations selected for the final printability assessment.

**FIGURE 4:**
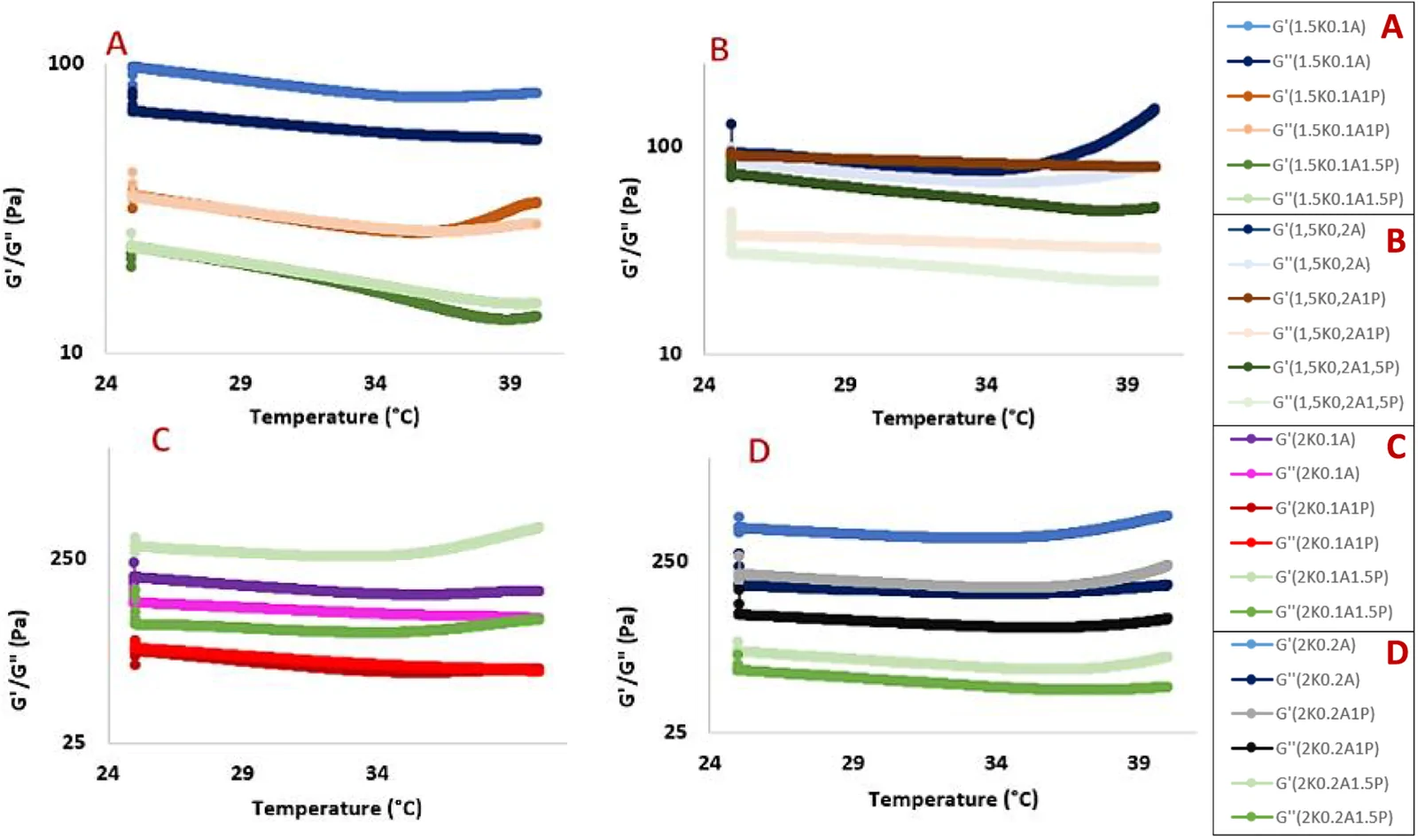
Temperature-dependent oscillatory rheological analysis of hydrogels made from konjac, agar and pectin, showing the storage modulus (G′) and loss modulus (G″) as a function of temperature from 25 °C to 40 °C. Panels **(A–D)** correspond to the series 1.5 K0.1A, 1.5 K0.2A, 2 K0.1A and 2 K0.2A, respectively. Moduli are expressed in Pa.

The oscillatory temperature sweeps shown in Figure 4 indicate predominantly solid-like viscoelastic behaviour under the tested conditions, with G′ remaining higher than G″ from 25 °C to 40 °C. This response shows that the formulations maintained elastic dominance throughout the evaluated temperature range at 1 Hz. Frequency-dependent behaviour was further examined in the four formulations selected for the final printability assessment.

**FIGURE 5:**
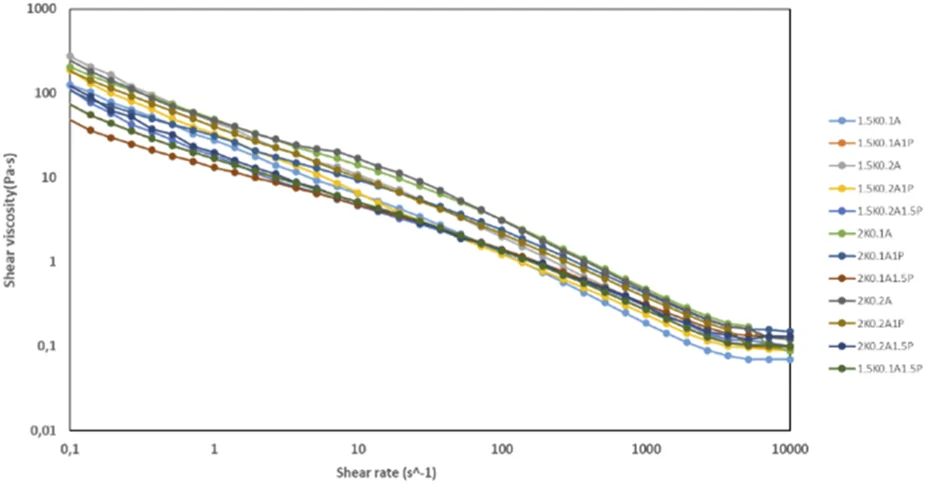
Apparent viscosity as a function of shear rate for all hydrogel formulations at 37 °C. All formulations showed shear-thinning behaviour, with decreasing viscosity as shear rate increased. Viscosity is expressed in Pa s and shear rate in s^-1^.

Viscosity *versus* shear rate analysis (Figure 5) confirms that all formulations exhibit non-Newtonian, pseudoplastic behaviour (Ardaneh et al., 2024). This behaviour is favourable for extrusion bioprinting, as it allows the viscosity to decrease under increasing shear rate, facilitating flow through the nozzle.

It was observed that formulations with higher agar concentration (such as 2K0.2A) had the highest viscosities at rest, which may increase the force required for extrusion. However, these same formulations showed a pronounced reduction in viscosity with increasing shear rate, reflecting a favourable shear-thinning response for extrusion. On the other hand, the incorporation of citrus pectin decreased the overall viscosity of the system, reducing its resistance to extrusion, although potentially compromising the mechanical stability of the material due to interference with the gelation interactions between agar and konjac (Luo et al., 2024).

The decrease in the elastic modulus observed after the incorporation of pectin may be attributed to a modification of the hydrogen-bonding network responsible for the gel’s rigidity. Pectin likely replaces part of the strong intermolecular interactions between agarose chains with weaker bonds formed between its own carboxyl and hydroxyl groups and the hydroxyl groups of agar. This partial substitution could reduce the crosslinking density and the internal cohesion of the polymer network, resulting in a more flexible structure with greater water mobility, which in turn leads to a lower storage modulus (G′). This behavior is consistent with the findings reported by Popov et al. (2022) (Popov et al., 2022), who demonstrated that the addition of pectin to agar gels decreases their hardness and stiffness by interfering with the formation of hydrogen bonds between agarose chains, confirming that pectin acts as a rheological modifier that softens the three-dimensional hydrogel network and reduces its mechanical strength.

These rheological results underline the need to balance the proportions of the components to obtain a hydrogel with suitable properties in both fluidity and stiffness. Formulations such as 2 K0.1A and 2K0.1A1P showed an adequate balance between stiffness, modulated by the agar content, and flowability, favoured by the presence of pectin. This balance may support extrusion while preserving the deposited geometry, although longer-term shape retention requires additional experimental validation. In contrast, stiffer formulations such as 2 K0.2A, while offering higher structural strength, may require adjustments in printing parameters to avoid extrusion problems.

The pseudoplastic behaviour common to all formulations supports their potential suitability for extrusion bioprinting applications, particularly from the point of view of flow through the nozzle under shear.

Taken together, these results position the studied hydrogels as promising alternatives for the development of functional materials in 3D bioprinting, highlighting the versatility of natural systems based on konjac, agar and pectin to adjust properties according to the specific requirements of each application. At this stage, the rheological data support the classification of the formulations as shear-thinning hydrogels with predominantly elastic behaviour under the tested conditions. Detailed frequency-dependent and recovery behaviour of the four selected formulations is presented in Section 3.6, while long-term structural stability will require specific time-dependent and stability studies.

### 3.3 Printability

#### 3.3.1 Extrusion force assay

During the extrusion force assay, the resistance of each hydrogel formulation to plunger displacement was measured under identical cartridge, nozzle, temperature and crosshead-speed conditions. The force required to initiate and maintain stable flow was used as a comparative indicator of resistance to flow among formulations.

Table 3 shows the resistance to flow of each hydrogel formulation, expressed as the minimum force required to obtain stable extrusion under the selected testing conditions. In the 1.5K0.1A series, the resistance to flow ranged from 9.4 to 13.6 N, with pectin-containing formulations showing lower values than the pectin-free formulation. This suggests that pectin reduced the resistance to extrusion, probably by softening the polymeric network and increasing the flowability of the formulation.

**TABLE 3:** Resistance to flow, expressed as the minimum force required for stable extrusion, for each hydrogel formulation under the selected testing conditions.

Hydrogel	Resistance to flow in the 10 mL syringe (N)
1.5K0.1A	13.6
1.5K0.1A1P	9.8
1.5K0.1A1.5 P	9.4
1.5K0.2A	32
1.5K0.2A1P	19
1.5K0.2A1.5 P	10.3
2K0.1A	19.5
2K0.1A1P	12.5
2K0.1A1.5 P	9.3
2K0.2A	34.6
2K0.2A1P	18.9
2K0.2A1.5 P	13.3

When the agar concentration was increased to 0.2% in the 1.5 K series, the pectin-free formulation showed a higher resistance to flow, reaching 32.0 N for 1.5K0.2A. However, the incorporation of pectin reduced this value to 19.0 N for 1.5K0.2A1P and 10.3 N for 1.5K0.2A1.5 P. This trend indicates that the effect of pectin on extrusion resistance was more evident in agar-richer formulations (Fu et al., 2021), (Ramesh et al., 2021).

A similar behaviour was observed in the 2 K series. The highest resistance to flow was recorded for 2 K0.2A, with 34.6 N, whereas the addition of pectin reduced the required force to 18.9 N for 2K0.2A1P and 13.3 N for 2K0.2A1.5 P. Therefore, increasing agar concentration generally increased the resistance to flow, while increasing pectin concentration reduced the force required for extrusion. These results are consistent with the rheological behaviour described previously, where agar contributed to higher stiffness and viscosity, whereas pectin acted as a softening component that facilitated extrusion.

The force measurements provided a comparative ranking of the resistance to flow of the different formulations under the selected testing conditions. The formulations were subsequently printed using the BIO X bioprinter, with pneumatic pressure adjusted independently for each formulation to obtain continuous deposition through the selected 22G nozzle. Overall, the extrusion force assay showed formulation-dependent differences in resistance to flow and highlighted the opposite effects of agar and pectin on extrusion-related behaviour.

Although shear stress is a relevant parameter in extrusion-based bioprinting, it was not directly calculated because the real volumetric flow rate through the conical nozzle was not measured. Accurate shear-stress estimation would require direct measurement of the flow rate, effective nozzle geometry and pressure drop for each formulation. Therefore, resistance to flow, apparent viscosity and filament-deposition behaviour were used as complementary comparative indicators of extrusion-related performance. Future studies will include direct flow-rate measurements and shear-stress estimation, particularly for cell-laden printing.

#### 3.3.2 Collapse test

The collapse test allows the evaluation of the ability of hydrogels to form unsupported bridges between pillars, simulating the construction of three-dimensional structures without auxiliary support. This property is relevant to short-term geometric retention during layer-by-layer printing. In the present study, this assay was interpreted as a comparative indicator of short-term filament stability after deposition, rather than as a direct prediction of long-term shape retention or creep behaviour.

The test results (Table 4) indicate that the formulations belonging to the 1.5 K series generally presented lower bridge-forming capacity than the 2 K series. Within this group, 1.5K0.1A was able to generate bridges between the eight pillars, whereas the incorporation of pectin progressively reduced the number of supported bridges to six and five for 1.5K0.1A1P and 1.5K0.1A1.5 P, respectively. In the 1.5K0.2A series, all formulations showed limited collapse performance, supporting only two bridges. This suggests that, in these formulations, increasing the agar concentration did not necessarily improve the stability of the unsupported filaments, likely due to excessive stiffness or reduced flexibility. A possible explanation is that excessive stiffness, together with changes in polymer-network cohesion, may reduce the ability of the extruded filament to accommodate deformation while spanning unsupported gaps (Zhang et al., 2019).

**TABLE 4:** Apparent viscosity at 0.1 s^-1^, resistance to flow and collapse-test results for the evaluated hydrogel formulations.

Sample	Viscosity (Pa·s)	Resistance to flow (N)	Collapse test (supported pillars)
1.5K0.1A	126.3	13.6	8
1.5K0.1A1P	74	9.8	6
1.5K0.1A1.5 P	60	9.4	5
1.5K0.2A	276	32	2
1.5K0.2A1P	189.6	19	2
1.5K0.2A1.5 P	113.3	10.3	2
2K0.1A	206.5	19.5	8
2K0.1A1P	109.6	12.5	7
2K0.1A1.5 P	48.3	9.3	5
2K0.2A	245.7	34.6	8
2K0.2A1P	183.7	18.9	8
2K0.2A1.5 P	121.8	13.3	5

In contrast, the 2 K series formulations obtained more favourable collapse-test results. Three formulations, 2 K0.1A, 2 K0.2A and 2K0.2A1P, were able to generate bridges between the eight pillars, indicating improved short-term filament stability after extrusion. Other formulations in this series supported between five and seven pillars, suggesting that the increase in konjac concentration contributed positively to bridge formation. This improvement may be related to higher viscosity and greater internal cohesion of the hydrogel network, which can promote the formation of more stable unsupported filaments (Zhang et al., 2019), (Hernández Velasco, 2004). However, the results also show that collapse behaviour was not determined solely by viscosity, since formulations with different viscosity values exhibited comparable bridge-forming capacity. Therefore, filament stability appears to depend on a balance between viscosity, stiffness, flow resistance and internal cohesion of the hydrogel network.

These findings underline the importance of component composition and ratio on the short-term structural performance of the extruded hydrogels. The best-performing formulations showed greater short-term resistance to filament deformation under the tested collapse conditions, suggesting a favourable balance between stiffness and flexibility. Nevertheless, these results should be interpreted as comparative collapse-test outcomes and not as complete validation of printability for complex three-dimensional constructs.

Table 4 summarises the results obtained, correlating viscosity, flow resistance and collapse capacity for each formulation. Overall, the 2 K formulations showed better bridge-forming performance than the 1.5 K formulations, which justified their selection for subsequent printability-related assays (Heidenreich et al., 2020).

The results shown in Table 4 indicate that, within comparable formulation series, higher viscosity was generally associated with higher resistance to flow. However, this relationship was not strictly linear across all formulations, indicating that additional factors, such as polymer ratio, network cohesion and filament flexibility, also influenced extrusion and collapse behaviour. The incorporation of pectin generally reduced both viscosity and resistance to flow, particularly at the highest pectin concentration, supporting its role as a softening component within the konjac-agar network.

#### 3.3.3 Relative self-supporting index

The relative self-supporting index was evaluated only for the selected 2 K formulations, which showed the most favourable collapse-test behaviour. This assay was used as a comparative indicator of resistance to vertical deformation under compression, rather than as a direct prediction of the maximum number of printable layers.

The mean compressive forces ranged from 0.02 to 0.05 N at 20% deformation and from 0.04 to 0.13 N at 40% deformation (Table 5). For all formulations, the force increased when the imposed deformation was increased from 20% to 40%.

**TABLE 5:** Compressive force and relative self-supporting index of the selected 2 K hydrogel formulations.

Hydrogel	Compressive force at 20% deformation (N)	Compressive force at 40% deformation (N)	Relative self-supporting index (37 °C)	
			20%	40%
2K0.1A	0.050 ± 0.004	0.13 ± 0.01	20	52
2K0.1A1P	0.030 ± 0.001	0.04 ± 0.01	12	16
2K0.1A1.5P	0.030 ± 0.003	0.08 ± 0.02	12	32
2K0.2A	0.040 ± 0.001	0.11 ± 0.02	16	44
2K0.2A1P	0.020 ± 0.003*	0.04 ± 0.05	8	16
2K0.2A1.5P	0.030 ± 0.005	0.08 ± 0.05	12	32

* The mean value of 0.020 N coincides with the lower limit of the ±0.5% load-measurement accuracy range of the 5 N load cell used in this assay.

As reported by other authors, these parameters probe different deformation mechanisms. Flow resistance describes the behaviour of the material during extrusion-related deformation, whereas compressive resistance reflects the ability of the hydrogel network to withstand vertical loading and large deformations. A similar lack of direct correlation between rheological and compressive properties has been reported by Hossain et al. (Hossain et al., 2021) and Stojkov et al. (Stojkov et al., 2021).

The relative self-supporting index showed a formulation-dependent numerical pattern. At 20% deformation, 2K0.1A presented the highest mean index value, 20, followed by 2 K0.2A, with an index of 16. The pectin-containing formulations showed lower values, ranging from 8 to 12. At 40% deformation, 2K0.1A again showed the highest mean index, 52, followed by 2 K0.2A, with an index of 44. Formulations containing 1.5% pectin showed intermediate values of 32, whereas 1% pectin formulations showed lower values of 16.

The pectin-free formulations, particularly 2 K0.1A, showed numerically higher compressive-force values and relative self-supporting indices. This pattern was interpreted as a descriptive trend, since small differences and comparisons involving overlapping experimental dispersion should be interpreted cautiously.

These results underline the importance of understanding and optimizing the composition of hydrogels for specific applications. In particular, 2K0.1A showed numerically the most favourable combination of collapse performance and relative resistance to vertical compression under the tested conditions, whereas 2K0.2A1P combined good collapse behaviour with lower extrusion resistance than 2K0.2A. These findings reinforce the need to identify balanced formulations according to the specific mechanical and printing requirements of each application, rather than relying on a single printability parameter.

Values are expressed as mean ± SD, from three independent measurements. The relative self-supporting index was calculated by dividing the compressive force recorded at each deformation level by the estimated weight of one deposited hydrogel layer, assumed to be 0.0025 N. The relative self-supporting index was calculated from the mean compressive force recorded at each deformation level and was used as a descriptive comparative parameter. This value was used only as a comparative index and should not be interpreted as an absolute prediction of the maximum number of printable layers.

#### 3.3.4 Line study

The line study was performed to analyse the homogeneity and dimensional regularity of the extruded filaments, which are relevant parameters for extrusion-based printing when continuous and reproducible hydrogel deposition is required. Through morphometric analysis of the extruded filaments, the minimum and maximum height values along the filament profile, together with the mean filament height and estimated area, were evaluated for each formulation (Table 6).

**TABLE 6:** Dimensional analysis of extruded hydrogel filaments. Minimum and maximum values correspond to the extreme values measured along the filament profile. Mean height and mean estimated area are expressed as mean ± SD from the measurements obtained along the analysed filaments.

​	Min (mm)	Max (mm)	Mean (mm) ± SD	Area min (mm^2^)	Area max (mm^2^)	Average (mm^2^) ± SD
2K0.1A	0.76	1.05	0.91 ± 0.23	8.81	11.46	10.14 ± 0.23
2K0.1A1P	1.43	1.57	1.50 ± 0.10	14.00	16.00	15.00 ± 0.95
2K0.1A1.5P	0.99	1.13	1.06 ± 0.06	8.15	9.24	8.70 ± 0.44
2K0.2A	1.10	1.35	1.23 ± 0.06	12.00	15.50	13.75 ± 0.76
2K0.2A1P	0.81	0.90	0.86 ± 0.02	7.28	7.74	7.51 ± 0.16
2K0.2A1.5P	1.74	1.96	1.85 ± 0.05	12.71	13.80	13.26 ± 0.91

The results showed formulation-dependent differences in filament dimensions and regularity. The analysed formulations generated continuous filaments under the selected printing conditions, although differences were observed in filament height and estimated area. For example, 2 K0.1A and 2K0.2A showed relatively broad height ranges, whereas pectin-containing formulations such as 2K0.2A1P showed a narrower height range. These differences may be related to the combined effect of hydrogel stiffness, flowability and resistance to extrusion, which can influence filament spreading and dimensional regularity after deposition.

Formulations such as 2K0.1A1.5 P showed moderate variability in both filament height and estimated area, indicating a relatively uniform extrusion profile under the tested conditions. However, these results should be interpreted as a comparative assessment of line uniformity and not as a complete validation of three-dimensional printing accuracy.

The analysis suggests that while high agar content may increase filament stiffness, its use without pectin does not necessarily guarantee greater filament uniformity. Pectin, while reducing stiffness, may improve the flowability of the system and contribute to a more regular extrusion profile in some formulations. This behaviour reinforces the importance of achieving a balance between stiffness and flowability in the design of hydrogels for extrusion-based bioprinting, adapting their rheological properties to the specific structural requirements of each application (Paxton et al., 2017).

**FIGURE 6:**
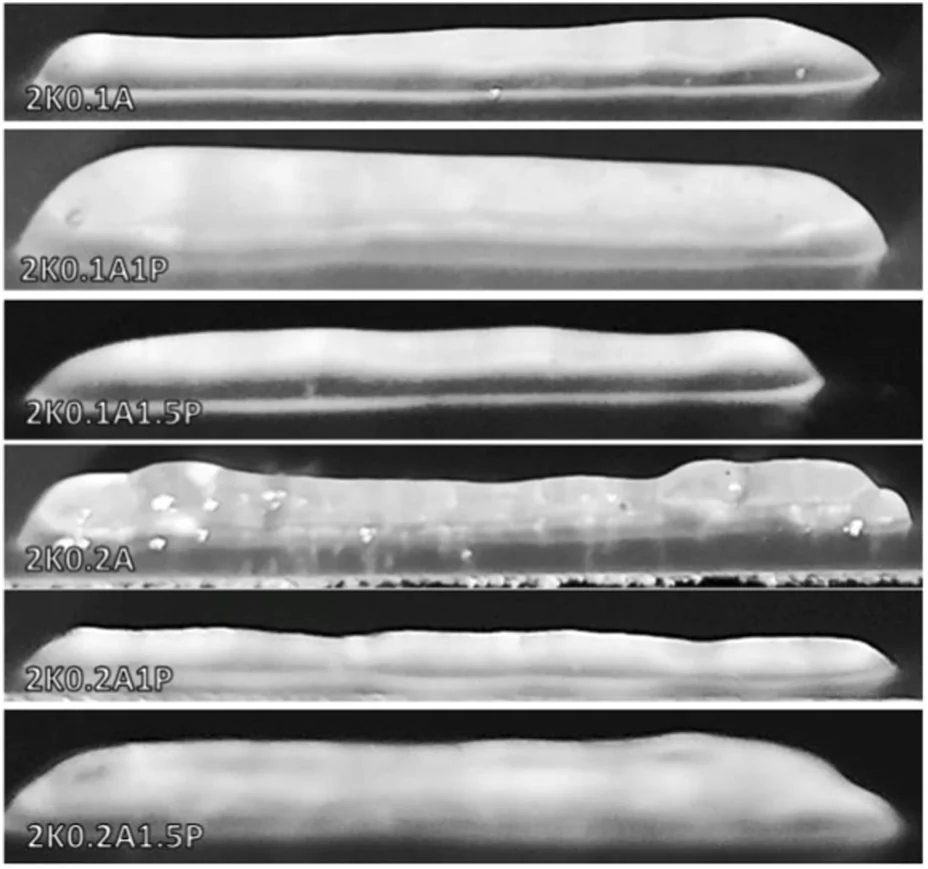
Filaments of the formulations to observe the homogeneity of their extrusion.

Therefore, the line study (Figure 6) shows that selected formulations can generate continuous and geometrically controlled filaments under the tested printing conditions. Among them, some pectin-containing formulations generally showed more regular filament deposition, whereas pectin-free and agar-richer formulations tended to show greater dimensional variability. These observations were used together with the extrusion-force, collapse and relative self-supporting assays to identify the most balanced formulations for subsequent printability evaluation.

### 3.4 Apparent metabolic activity and cell morphology in selected formulations

**FIGURE 7:**
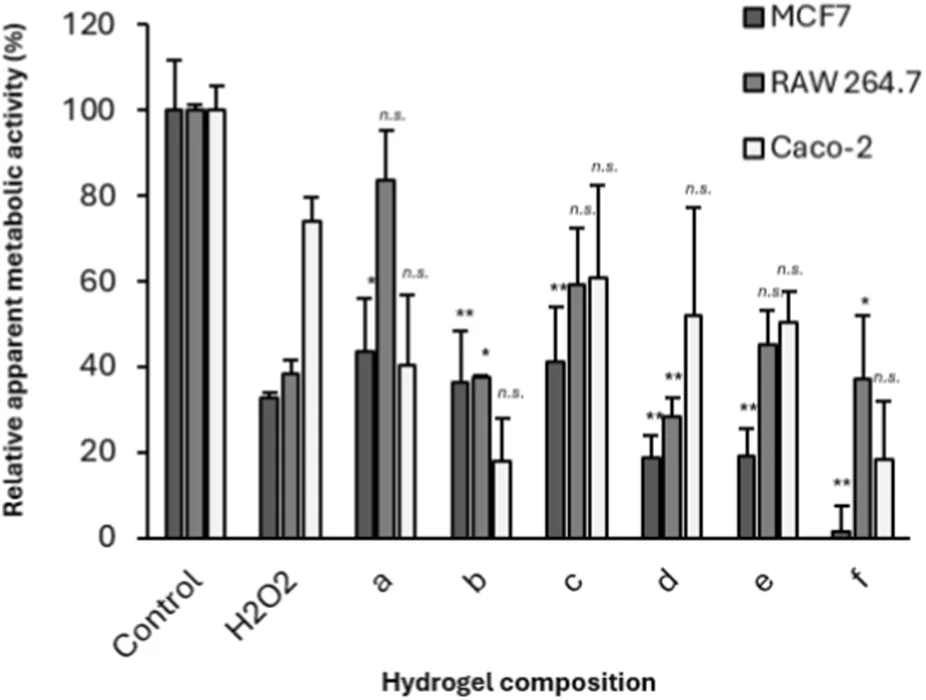
Cell-dependent effects of selected hydrogel formulations on apparent metabolic activity. MTS metabolic activity was measured at 24 h post-seeding and expressed as a percentage relative to each cell-type control, set as 100%. a-f indicate different hydrogel formulations: **(a)** 2 K0.1A, **(b)** 2K0.1A1P, **(c)** 2K0.1A1.5 P, **(d)** 2 K0.2A, **(e)** 2K0.2A1P, and **(f)** 2K0.2A1.5 P. Data are presented as mean ± standard deviation of triplicate values from at least two biological replicates (n ≥ 6). Statistical significance was analysed separately for each cell line using one-way ANOVA followed by Dunnett’s multiple-comparison test against the corresponding cell-type control. *p < 0.05; **p < 0.01; n. s., non-significant. H_2_O_2_ was included as a positive control and was not included in the statistical comparisons.

Following printability optimization, we evaluated the biological response to the selected formulations. First, we performed direct-contact MTS assays using six hydrogel formulations. To account for cell diversity in future applications, we extended our studies to 3 cell lines representing different cell lineages, including MCF-7 cells (mammary epithelium), RAW 264.7 cells (immune system), and Caco-2 cells (colorectal epithelium). At 24 h post-seeding, we observed a cell-dependent response to the hydrogels, which varied according to both formulation and cell lineage (Figure 7).

All selected formulations reduced the MTS signal in MCF-7 cells, consistent with the response observed for agar-containing conditions in the individual-component screening. In MCF-7 cells, formulations containing 0.2% agar showed a stronger reduction in MTS signal than those containing 0.1% agar; however, this trend should be interpreted cautiously because the assay reflects apparent metabolic activity in direct contact with the hydrogel and may also be influenced by cell adhesion and aggregation. In the case of RAW 264.7 cells, we observed a formulation-dependent response, with reductions in the MTS signal varying according to the combined presence of agar and pectin. Caco-2 cells also showed a formulation-dependent response, although the magnitude of the reduction differed among compositions. Future studies will evaluate whether additional variables related to the bioprinting process, such as temperature, pressure, speed, and direct cell encapsulation, may have an additional impact on cellular response. As an internal control in our biological assays, we used H_2_O_2_ as a cytotoxic chemical exemplar widely reported as a cell death-inducing agent.

It should be noted that, in the present experimental design, cells were seeded directly on top of the hydrogel layers and MTS absorbance was measured in wells containing both the hydrogel and the cells. Under these conditions, matrix-related interference cannot be completely excluded. In particular, the hydrogel network may affect reagent diffusion, soluble formazan recovery, optical path length, or light scattering during absorbance measurement. In addition, differences in cell adhesion, spreading, and aggregation on hydrogel surfaces may alter the metabolic signal independently of actual membrane damage. Therefore, MTS values should not be interpreted as a direct absolute measurement of viable cell number, but rather as an indicator of apparent metabolic activity under each experimental condition. As a result, the MTS assay may underestimate cell viability in some formulations. For this reason, metabolic activity data were interpreted as a preliminary quantitative screening and complemented with confocal microscopy using Hoechst 33342 and propidium iodide staining, which provided additional information on cell distribution, morphology, and membrane integrity.

**FIGURE 8:**
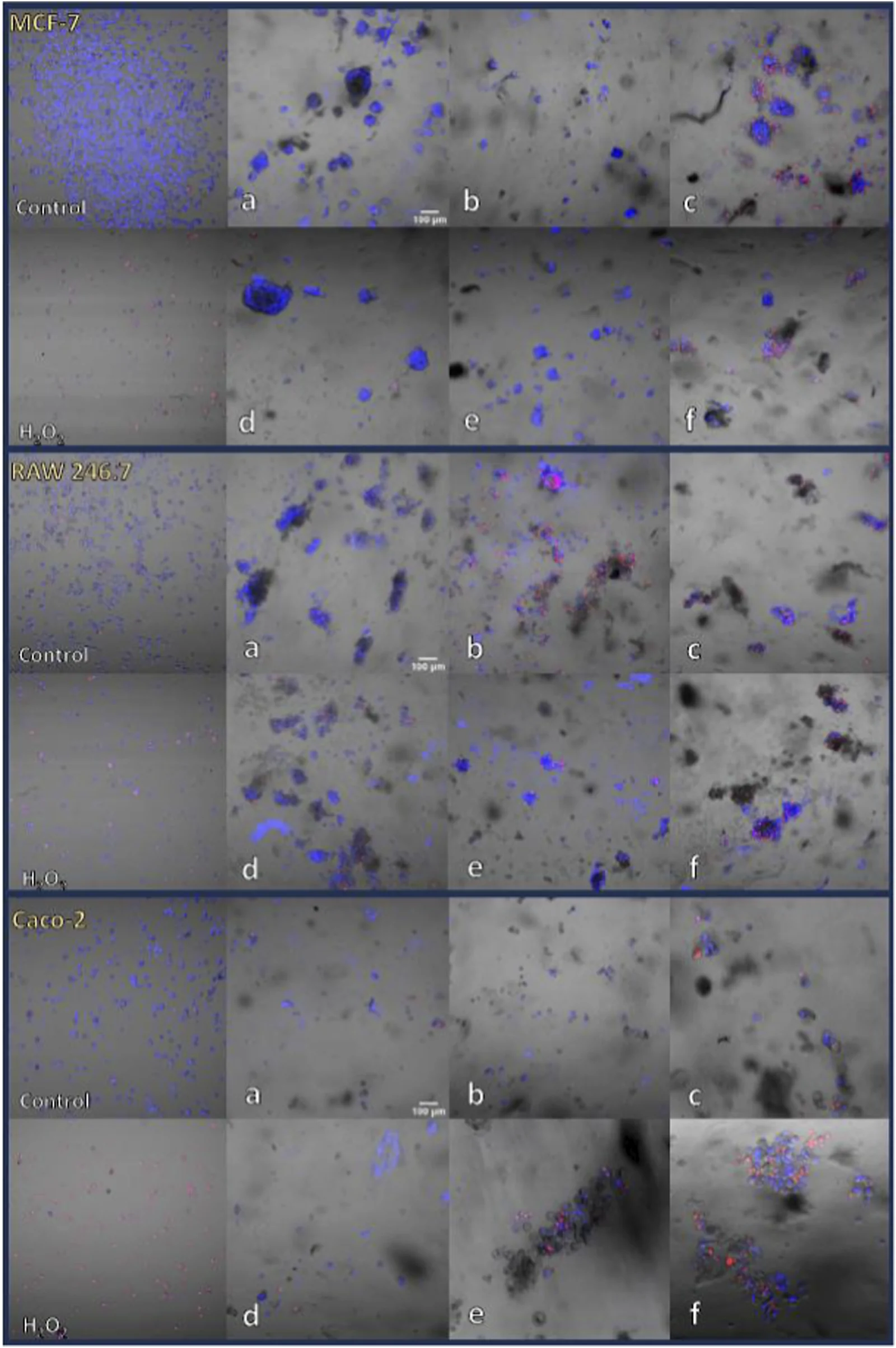
Qualitative confocal assessment of cell morphology, distribution, and membrane integrity after 24 h of culture on selected hydrogel formulations. Hoechst 33342 was used to identify cell nuclei, whereas propidium iodide was used to detect cells with compromised membrane integrity. a–f indicate different hydrogel formulations: **(a)** 2 K0.1A, **(b)** 2K0.1A1P, **(c)** 2K0.1A1.5 P, **(d)** 2 K0.2A, **(e)** 2K0.2A1P, and **(f)** 2K0.2A1.5 P. Images are representative fields and were interpreted qualitatively; no quantitative image analysis was performed.

The apparent discrepancy between the marked reduction in MTS signal and the limited PI staining observed qualitatively in several konjac-agar formulations (Figure 8) indicates that the metabolic readout cannot be interpreted as an equivalent loss of viable cells. Instead, the results are consistent with a combination of reduced cell adhesion, spheroid-like aggregation and matrix-related optical or diffusional interference. This interpretation is supported by previous studies showing that MCF-7 cells can proliferate and form colonies in 0.5% agar and that 0.25% agar can promote multicellular spheroid formation (Schmid et al., 2020), (Chen et al., 2022). Therefore, the present data do not demonstrate either extensive cytotoxicity or definitive cytocompatibility, and both possibilities require confirmation using matrix-independent assays.

Although direct-contact assays are useful for screening the cellular response to multiple hydrogel formulations, the present approach should not be considered equivalent to a full cytotoxicity assessment according to ISO 10993–5. Therefore, the results are interpreted here as an initial safe(r)-by-design screening of cellular response under direct-contact culture conditions. MTS-based screening assays are useful for the quantitative screening of multiple experimental conditions. However, they do not provide sufficient information on other relevant parameters for hydrogel development, such as cell morphology, spatial distribution, aggregation, or membrane integrity. Therefore, confocal microscopy was used to further assess the impact of the selected hydrogel formulations on cell behaviour (Figure 8).

Specifically, we stained cells with a combination of Hoechst 33342, marking cell nuclei, and propidium iodide (PI), selectively staining cells with compromised viability. To encompass the wide range of potential applications of hydrogels, we selected cell lines representing different lineages, including MCF-7, RAW 264.7, and Caco-2 cells. These models exhibit varying levels of metabolic competence, migratory capacity, and growth characteristics.

Qualitative inspection revealed changes in cell morphology and distribution in the presence of the tested hydrogels. Compared to non-exposed cells, the presence of hydrogels induced a spheroid-like distribution of MCF-7 and RAW 264.7 cells. This effect was less profound in Caco-2 cells, which appeared more evenly distributed in some formulations. In spite of this phenotypic effect, PI staining appeared limited in several konjac-agar-containing formulations, suggesting that the reduced MTS signal was not consistently accompanied by evident membrane damage. However, formulations containing higher pectin concentrations showed a tendency toward increased PI staining in some cell lines, particularly MCF-7 and RAW 264.7 cells. Because PI-positive cells were not quantified in this study, this observation should be interpreted qualitatively and not as a definitive measure of apoptosis or cell death. These results complement those obtained using traditional metabolic activity assays. Overall, the combined MTS and confocal observations suggest that the biological response to these hydrogels may involve both changes in apparent metabolic activity and changes in cell-material interactions, including adhesion, spreading, and aggregation. Further experiments, including quantitative live/dead image analysis and extract-based cytotoxicity testing, will be required to distinguish genuine cytotoxicity from reduced adhesion or assay-related effects.

The observed phenotypic and metabolic effects may extend beyond biochemical properties of individual hydrogel components. For example, the electrically neutral smooth surface provided by the agar has been shown to inhibit receptor binding and cell adhesion (Spitz-Argolo et al., 2025), a critical factor for cell viability. In addition, cells cultured on poorly adhesive or non-adherent substrates may form aggregates rather than remaining dispersed, which promotes intercellular communication and survival through gap junctions and the secretion of survival factors (do Amaral et al., 2011), (Langer et al., 2019). Furthermore, three-dimensional (3D) spheroid organisation enhances cell viability by providing cell-cell interactions and an environment more akin to *in vivo* conditions (do Amaral et al., 2011). In this context, it has been reported that breast cancer cells bioprinted in 3D environments activate phosphorylation mechanisms of proteins involved in apoptosis resistance, suggesting an adaptive advantage in such systems (Campbell et al., 2019).

As agar lacks specific anchorage sites for mammalian cells, cell viability in hydrogels containing this polymer depends on the ability of cells to survive in a non-adherent environment. It has been demonstrated that MCF-7 cells can grow in both Matrigel and 0.5% (w/v) agar, although the colonies formed in agar are smaller due to the absence of adhesion to the matrix (Schmid et al., 2020). Similarly, 0.25% (w/v) agar has been used to generate multicellular spheroids for nanoparticle permeability studies, supporting its application in 3D cultures (Chen et al., 2022). These previous studies support the interpretation that a reduced MTS signal on agar-containing surfaces may partly reflect altered adhesion and spheroid-like organisation rather than direct cytotoxicity alone.

The incorporation of pectin modified the cellular response of the konjac-agar formulations. However, the available data do not allow this effect to be attributed to intrinsic pectin cytotoxicity. Changes in hydrogel structure, cell adhesion, nutrient diffusion and matrix-related interference with the MTS assay may also have contributed to the observed differences.

Overall, these findings demonstrate that the cellular response depended on both hydrogel composition and cell type. Formulations containing 0.1% agar and 1% pectin generally maintained a higher apparent metabolic signal than formulations with higher agar or pectin concentrations. However, the confocal observations indicate that the reduction in MTS signal was not consistently accompanied by extensive membrane damage and may partly reflect altered cell adhesion, aggregation and matrix-related assay interference. Therefore, the selected formulations should be considered candidates for further optimisation rather than confirmed cytocompatible bioinks. Quantitative live/dead analysis, extract-based cytotoxicity assays and cell-laden printing experiments will be required for definitive biological validation.

### 3.5 Choice of selected hydrogel formulations based on printability-related performance

After the biological screening and the previous printability-related assays, four selected formulations were chosen for the final shape-fidelity evaluation: 2 K0.1A, 2K0.1A1P, 2 K0.2A and 2K0.2A1P. These formulations were selected because they showed the most balanced behaviour among extrusion resistance, collapse performance, relative self-supporting index and preliminary cellular response under the tested conditions.

**FIGURE 9:**
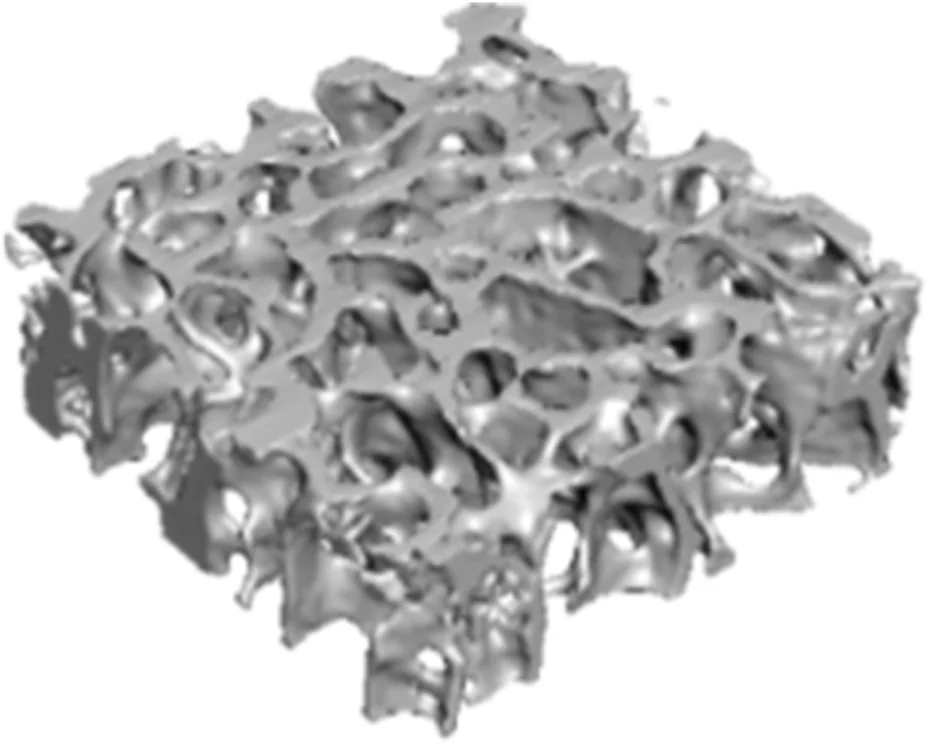
Trabecular bone-inspired CAD model used as a qualitative complex-geometry printing example.

**FIGURE 10:**
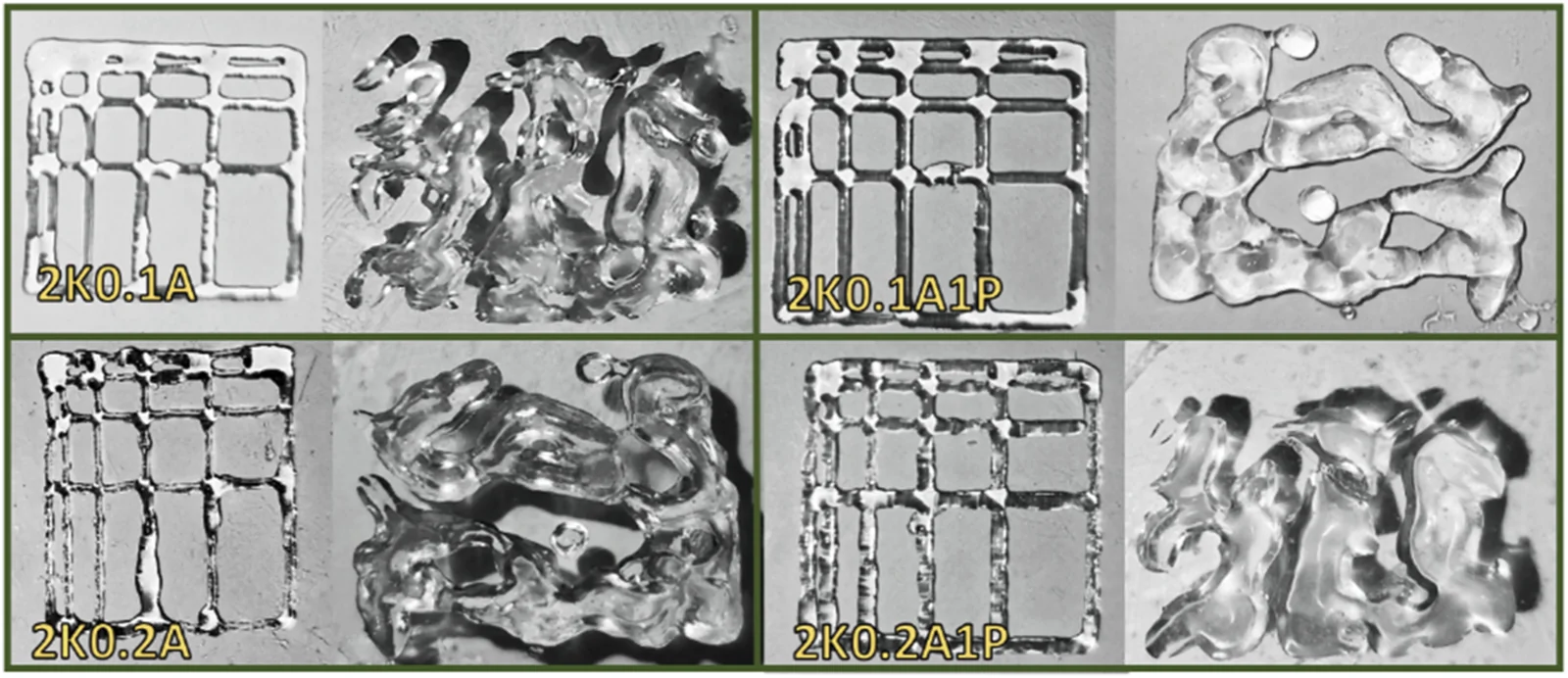
Final printability evaluation of the four selected hydrogel formulations. Left panels: printed grid structures used for quantitative shape-fidelity analysis. Right panels: qualitative trabecular bone-inspired constructs printed with each formulation to illustrate multilayer deposition behaviour.

For this purpose, grid-like structures of different dimensions were printed to evaluate the geometric accuracy of each selected hydrogel formulation. In parallel, a trabecular bone-inspired model, based on the CAD model shown in Figure 9, was printed as a qualitative multilayer printing example. After printing the grids (Figure 10, left panels), top-view images were scaled using a known reference distance and analysed in AutoCAD. For each measurable pore, the internal area was compared with the corresponding theoretical area defined in the CAD model, and the relative pore area was calculated as described in Section 2.3.5.

In addition to grid analysis, the trabecular bone-inspired model was used only as a qualitative demonstration (Figure 10, right panels), highlighting the potential of the formulations to reproduce more complex geometries. This model was not used for quantitative shape-fidelity analysis, since its geometry introduces additional variables related to multilayer deposition, filament stacking and local deformation.

**FIGURE 11:**
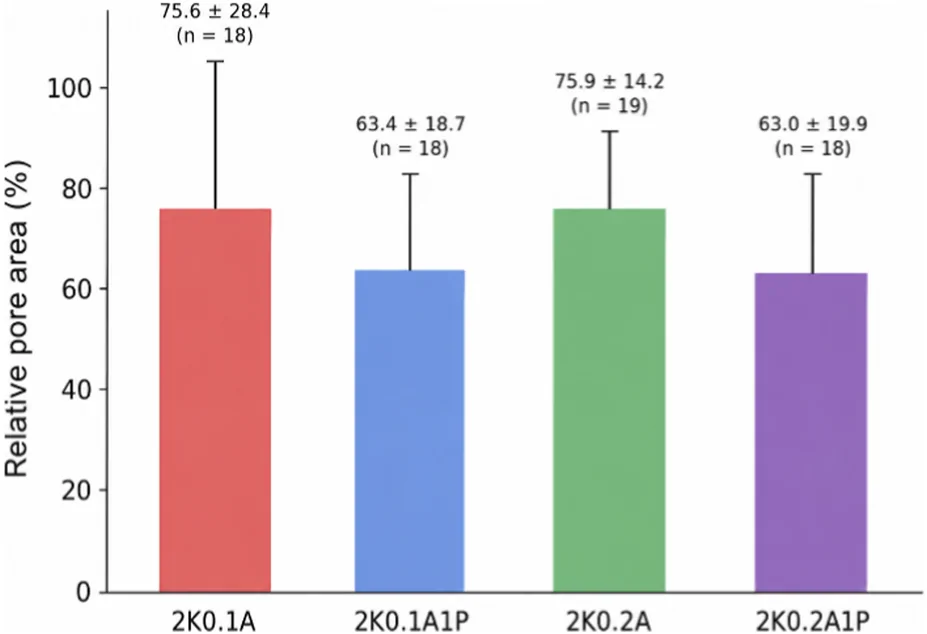
Relative pore area of the printed pore geometries for the four selected hydrogel formulations. Relative pore area was calculated by dividing the measured internal pore area by the corresponding theoretical CAD area and multiplying by 100. Bars represent mean values and error bars represent SD of the measurable pore geometries: n = 18 for 2 K0.1A, n = 18 for 2K0.1A1P, n = 19 for 2K0.2A and n = 18 for 2K0.2A1P. Non-assessable pore geometries were reported separately and excluded from the mean and SD calculation. The analysed pores had different theoretical sizes and were obtained from one independent printed grid per formulation; therefore, the SD represents within-grid dispersion among pore geometries of different sizes rather than repeatability among identical pores or independent printing experiments.

The quantitative area-based pore analysis is summarised in Figure 11 and Table 7. The mean relative pore area was 75.6% ± 28.4% for 2 K0.1A, 63.4% ± 18.7% for 2K0.1A1P, 75.9% ± 14.2% for 2 K0.2A and 63.0% ± 19.9% for 2K0.2A1P. Of the 20 designed pore geometries, 18 were measurable for 2 K0.1A, 18 for 2K0.1A1P, 19 for 2 K0.2A and 18 for 2K0.2A1P, corresponding to measurable-pore rates of 90%, 90%, 95% and 90%, respectively.

**TABLE 7:** Summary of the area-based analysis of the printed pore geometries. Values are expressed as mean ± SD of the measurable pore geometries.

Formulation	Designed pores	Measurable pores	Non-assessable pores	Measurable-pore rate (%)	Relative pore area (%)
2K0.1A	20	18	2	90	75.6 ± 28.4
2K0.1A1P	20	18	2	90	63.4 ± 18.7
2K0.2A	20	19	1	95	75.9 ± 14.2
2K0.2A1P	20	18	2	90	63.0 ± 19.9

The smallest designed pore geometry, corresponding to pore 20 and a theoretical area of 0.03 mm^2^, could not be reliably formed or measured in any of the four formulations under the selected printing conditions. Additional non-assessable geometries were observed for pore 19 in 2 K0.1A, pore 15 in 2K0.1A1P and pore 16 in 2K0.2A1P. These cases were reported as NA and were excluded from the calculation of the mean relative pore area and SD but were retained when calculating the measurable-pore rate.

The relatively broad dispersion observed for some formulations was associated with the range of theoretical pore areas included in the analysis, which extended from 0.03 to 1.04 mm^2^. In general, the larger pore geometries showed measured areas closer to their theoretical values, whereas the smaller geometries showed greater deviations and, in some cases, did not present a sufficiently defined internal contour for reliable measurement. The SD therefore mainly reflects size-dependent variability among different pore geometries within the printed grid.

The pectin-free formulations showed descriptively higher mean relative pore-area values than the corresponding pectin-containing formulations. This trend was consistent with the qualitative observation of more clearly defined internal pore geometries in the pectin-free grids. In contrast, the pectin-containing formulations remained printable and produced continuous grid structures but showed greater geometric deviation, which may be associated with their lower extrusion resistance and greater spreading after deposition.

Taken together, the results indicate a composition-dependent balance between extrusion behaviour and preservation of the designed pore geometry. The pectin-free formulations combined higher mean relative pore-area values with higher extrusion resistance, whereas pectin-containing formulations showed lower resistance to flow but lower mean relative pore-area values. When considered together with the remaining rheological and printability-related assays, 2K0.1A showed one of the most balanced overall performance profiles under the selected experimental conditions.

### 3.6 Frequency-dependent and recovery behaviour of the selected formulations

**FIGURE 12:**
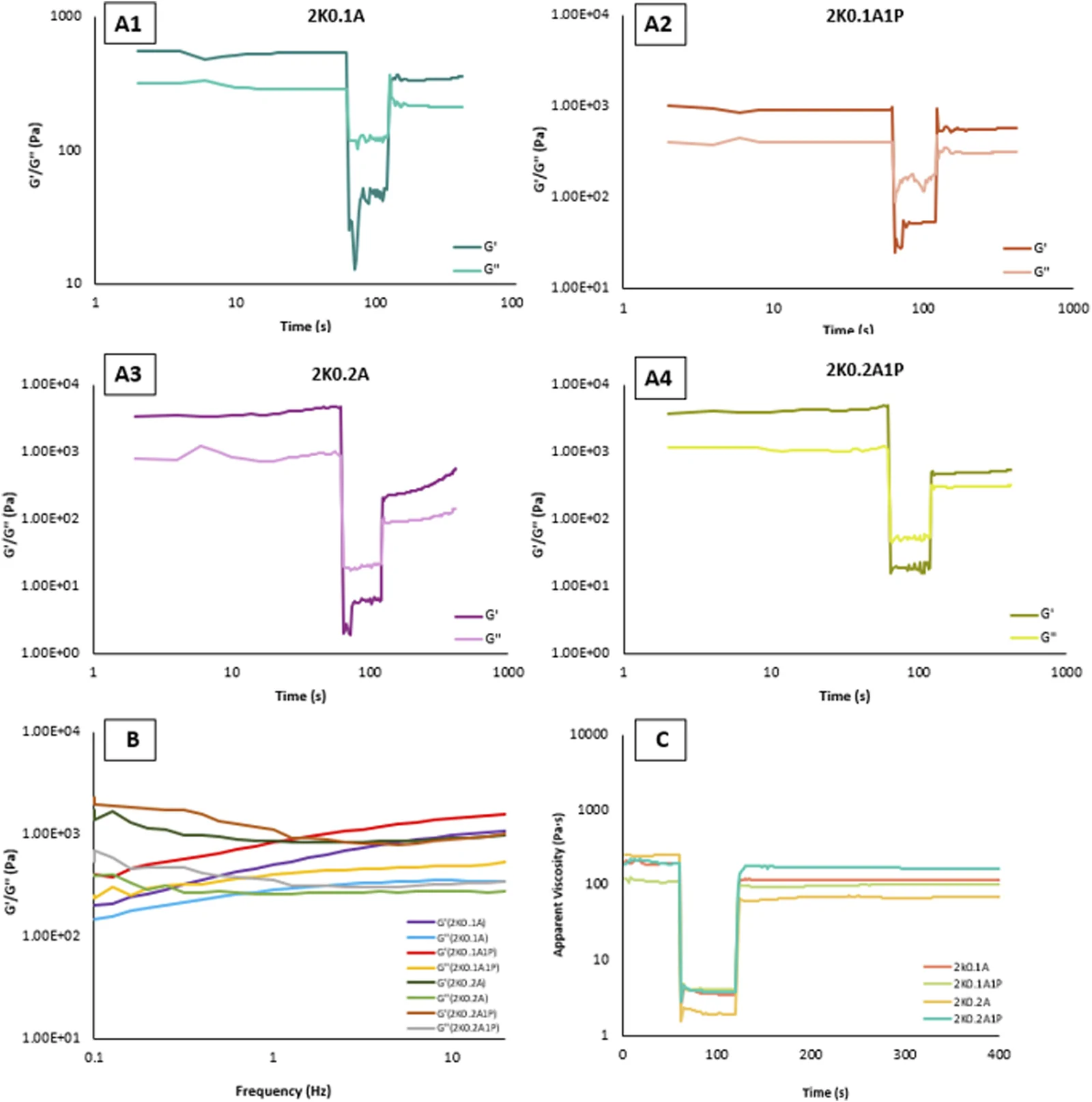
Rheological characterisation of the four selected hydrogel formulations at 37 °C. **(A1–A4)** Representative three-step oscillatory strain-recovery profiles of 2 K0.1A, 2K0.1A1P, 2 K0.2A and 2K0.2A1P, respectively. Measurements were performed at 1 Hz using 0.5% strain for 60 s, 300% strain for 60 s and 0.5% strain for 300 s. Storage modulus (G′) and loss modulus (G″) were recorded throughout the test. **(B)** Frequency-dependent storage and loss moduli of the four selected formulations measured from 0.1 to 20 Hz at 0.5% strain. **(C)** Complementary three-step shear-rate recovery profiles obtained at 0.1 s^-1^ for 60 s, 100 s^-1^ for 60 s and 0.1 s^-1^ for 280 s. Representative curves from one of the three independent measurements performed for each assay and formulation are shown.

Frequency sweep tests were performed on the four formulations selected from the printability-related assessment to examine their viscoelastic response over a broader range of oscillatory conditions (Figure 12B). For all four formulations, G′ remained higher than G″ throughout the analysed frequency range from 0.1 to 20 Hz, and no crossover between the moduli was observed. This response supports predominantly solid-like viscoelastic behaviour within the investigated frequency window and is consistent with the presence of a continuous physically associated network.

The magnitude and frequency dependence of the moduli differed among formulations. Both G′ and G″ varied across the analysed interval. Nevertheless, elastic dominance was maintained in all cases, including at the lowest analysed frequencies, where no transition towards predominantly viscous behaviour was detected. The absence of a G′-G″ crossover supports the persistence of gel-like viscoelastic behaviour over the timescales probed by the frequency sweep.

The recovery behaviour of the four selected formulations was evaluated using complementary oscillatory and shear-rate protocols (Figure 12A). During the initial 0.5% strain interval of the oscillatory test, G′ remained higher than G″ for all four formulations, indicating a predominantly elastic response within the linear viscoelastic region. Increasing the strain to 300% caused a marked decrease in G′ and an inversion of the relative moduli, with G″ exceeding G′ during the high-strain interval. This response confirms that the imposed deformation disrupted the initial solid-like viscoelastic behaviour.

When the strain was returned to 0.5%, G′ again exceeded G″ in all four formulations, demonstrating the short-term re-establishment of a predominantly elastic response after removal of the large deformation. The extent of modulus recovery was nevertheless formulation-dependent. The representative profiles of 2 K0.1A and 2K0.1A1P recovered a larger proportion of their initial G′ magnitude, whereas 2 K0.2A and 2K0.2A1P re-established G′ > G″ however, they remained below their initial storage modulus values during the recovery interval analysed. Therefore, the return to elastic behaviour did not necessarily imply a complete restoration of the initial modulus.

The complementary shear-rate test showed a marked decrease in apparent viscosity when the shear rate was increased, followed by formulation-dependent recovery after the initial low-shear condition was restored. Recovery was more pronounced for 2K0.1A1P and 2K0.2A1P, whereas 2K0.2A remained clearly below its initial apparent viscosity. These data describe the recovery of flow-related viscosity under low-shear measurement conditions. In contrast, the oscillatory profiles distinguish the re-establishment of a predominantly elastic response from viscosity recovery alone. Together, the two protocols provide complementary information on post-disruption behaviour, with G′ and G″ supplying the primary evidence of short-term recovery of solid-like viscoelastic behaviour.

## 4 Applications and future prospects

This study highlights the potential of natural hydrogels formulated from agar, konjac, and pectin for three-dimensional bioprinting. However, to move towards practical applications and incorporate a safe(r)-by-design approach, initial research steps should evaluate cell viability and functionality in structures printed with these hydrogels. Therefore, adapted formulations could be developed for specific applications such as bone, cartilage, and epithelial regeneration, taking advantage of their tunable mechanical and biological properties. Ensuring reproducibility, storage stability, and compatibility with large-scale bioprinting technologies is an essential step for their industrial implementation.

These hydrogels show great potential for applications in the fabrication of tissue scaffolds, bioprinted implants, and organoid models for pharmaceutical and biomedical research. In this context, recent reviews on natural biomaterials for 3D bioprinting emphasize that the exploitation of biopolymers derived from natural sources represents a promising strategy to advance regenerative medicine applications and support future industrial implementation (Mao et al., 2020).

## 5 Conclusion

In this study, hydrogel formulations based on agar, konjac and pectin were evaluated for future extrusion-based bioprinting applications. Their rheological and printability-related properties were assessed together with apparent metabolic activity and qualitative cell morphology under direct-contact conditions. This initial safe(r)-by-design screening allowed the identification of candidate formulations for further biological and printing-related validation.

Formulations with higher agar concentrations generally showed greater rheological stiffness, as reflected by their higher storage-modulus values. Across the four selected formulations, G′ remained higher than G″ from 0.1 to 20 Hz, with no crossover between the moduli, supporting predominantly solid-like viscoelastic behaviour consistent with a continuous physically associated network. After large-amplitude deformation, the four selected formulations re-established the condition G′ > G″, indicating recovery of a predominantly elastic response, although the initial modulus was only partially restored and the extent of recovery depended on hydrogel composition. The addition of pectin reduced low-shear viscosity and extrusion resistance, but was generally associated with lower hydrogel stiffness, lower mean relative self-supporting indices and lower mean relative pore-area values in the analysed grids.

Among the evaluated formulations, 2K0.1A showed the most balanced rheological and printability-related behaviour, combining moderate extrusion resistance, favourable collapse performance, the numerically highest mean relative self-supporting index and one of the highest mean relative pore-area values under the tested conditions. On the other hand, formulations such as 2 K0.2A, although mechanically stable, presented a lower filament homogeneity, which could limit their applicability in certain printing configurations.

The biological response varied according to both formulation and cell type. However, because the MTS signal may have been influenced by cell adhesion, aggregation and hydrogel-related assay interference, the present results cannot be interpreted as definitive evidence of either cytotoxicity or cytocompatibility. Further matrix-independent cytotoxicity assays and quantitative live/dead analyses are required before these formulations can be validated for cell-laden bioprinting applications.

Furthermore, by focusing on natural materials such as agar, konjac and pectin, this study explores a potentially sustainable material platform for extrusion-based bioprinting. However, its definitive biological suitability must be confirmed through additional matrix-independent and cell-laden validation studies.

## Data Availability

The original contributions presented in the study are included in the article/Supplementary Material, further inquiries can be directed to the corresponding author/s.
