# Supplementary material for: Initial safe(r)-by-design assessment of printable konjac-agar-pectin hydrogels for future extrusion-based bioprinting applications
Source: Front Bioeng Biotechnol. 2026 Aug 28;14:1900478. doi: 10.3389/fbioe.2026.1900478 (PMC13562252; doi:10.3389/fbioe.2026.1900478)
Supplement: Supplementary file 1 [file DataSheet1.pdf]

Supplementary Information

INITIAL SAFE(R)-BY-DESIGN ASSESSMENT OF PRINTABLE KONJAC-AGAR-PECTIN HYDROGELS FOR FUTURE EXTRUSION-BASED BIOPRINTING APPLICATIONS

Laura Mendoza-Cerezo[a,b], Francisco Inesta-Vaquera[b], Antonio Macías-García[a], Alfonso C. Marcos-Romero[a], Jesús M. Rodríguez-Rego[a*]*

[a] Departamento de Expresión Gráfica. Facultad de Ingeniería Industriales. Universidad de Extremadura. Avenida de Elvas, s/n. 06006-Badajoz. Spain.

[b] Departamento de Bioquímica, Biología Molecular y Genética. Facultad de Ciencias. Universidad de Extremadura. Avenida de Elvas, s/n. 06006-Badajoz. Spain

*jesusrodriguezrego@unex.es

*finestavaquera@unex.es

Contents of Supplementary Material

- **Table S1:** Quantitative pore-area analysis of printed grids for selected formulations (2K0.2A, 2K0.2A1P, 2K0.1A, 2K0.1A1P).

Table S1: Grid-Shape Fidelity and Pore Area Analysis

Detailed quantitative analysis of the 20 designed pore geometries obtained from printed grids using the four selected hydrogel formulations (2K0.2A, 2K0.2A1P, 2K0.1A, and 2K0.1A1P). The internal contour of each measurable pore was manually traced in AutoCAD to calculate the measured pore area and the corresponding relative pore area percentage.

2K0.2A			
Pore ID	Theoretical pore area (mm²)	Measured pore area (mm²)	Relative pore area (%)
1	1.04	0.92	88.46
2	0.83	0.70	84.14
3	0.62	0.45	72.12
4	0.42	0.31	74.57
5	0.21	0.10	48.08
6	0.52	0.45	86.52
7	0.42	0.32	76.92
8	0.31	0.20	64.08
9	0.21	0.12	57.72
10	0.10	0.08	76.92
11	0.26	0.24	92.31
12	0.21	0.17	81.73
13	0.16	0.14	89.74
14	0.10	0.09	88.55
15	0.05	0.03	48.08
16	0.13	0.11	84.55
17	0.10	0.09	86.96
18	0.08	0.07	83.33
19	0.05	0.03	57.69
20	0.03	NA	NA
**Measurable pores: 19/20**			
**Relative pore area: 75.9 ± 14.2%**			

2K0.2A1P			
Pore ID	Theoretical pore area (mm²)	Measured pore area (mm²)	Relative pore area (%)
1	1.04	0.87	83.65
2	0.83	0.63	75.73
3	0.62	0.50	80.13
4	0.42	0.31	74.57
5	0.21	0.06	28.85
6	0.52	0.41	78.83
7	0.42	0.31	74.52
8	0.31	0.23	73.69
9	0.21	0.17	81.77
10	0.10	0.05	48.08
11	0.26	0.20	76.92
12	0.21	0.14	67.31
13	0.16	0.11	70.51
14	0.10	0.07	67.37
15	0.05	0.01	26.92
16	0.13	NA	NA
17	0.10	0.05	48.31
18	0.08	0.04	53.85
19	0.05	0.01	23.08
20	0.03	NA	NA
**Measurable pores: 18/20**			
**Relative pore area: 63.0 ± 19.9%**			

2K0.1A			
Pore ID	Theoretical pore area (mm²)	Measured pore area (mm²)	Relative pore area (%)
1	1.04	0.87	83.65
2	0.83	0.68	81.74
3	0.62	0.54	86.54
4	0.42	0.34	81.79
5	0.21	0.13	62.50
6	0.52	0.42	80.75
7	0.42	0.34	81.73
8	0.31	0.27	86.51
9	0.21	0.16	76.96
10	0.10	0.15	144.23
11	0.26	0.05	19.23
12	0.21	0.16	76.92
13	0.16	0.14	89.74
14	0.10	0.10	96.25
15	0.05	0.05	96.15
16	0.13	0.05	38.43
17	0.10	0.04	38.65
18	0.08	0.03	38.46
19	0.05	NA	NA
20	0.03	NA	NA
**Measurable pores: 18/20**			
**Relative pore area: 75.6 ± 28.4%**			

2K0.1A1P			
Pore ID	Theoretical pore area (mm²)	Measured pore area (mm²)	Relative pore area (%)
1	1.04	0.90	86.54
2	0.83	0.75	90.16
3	0.62	0.44	70.51
4	0.42	0.30	72.17
5	0.21	0.10	48.08
6	0.52	0.40	76.91
7	0.42	0.30	72.12
8	0.31	0.22	70.49
9	0.21	0.14	67.34
10	0.10	0.02	19.23
11	0.26	0.17	65.38
12	0.21	0.13	62.50
13	0.16	0.09	57.69
14	0.10	0.09	86.62
15	0.05	NA	NA
16	0.13	0.05	34.59
17	0.10	0.06	57.97
18	0.08	0.05	64.10
19	0.05	0.02	38.46
20	0.03	NA	NA
**Measurable pores: 18/20**			
**Relative pore area: 63.4 ± 18.7%**			

NA, not assessable. Pore 20, corresponding to the smallest theoretical pore area (0.03 mm²), could not be reliably formed or measured in any formulation.
